# RIPK1-dependent necroptosis promotes vasculogenic mimicry formation via eIF4E in triple-negative breast cancer

**DOI:** 10.1038/s41419-023-05841-w

**Published:** 2023-05-22

**Authors:** Fan Li, Huizhi Sun, Yihui Yu, Na Che, Jiyuan Han, Runfen Cheng, Nan Zhao, Yuhong Guo, Chongbiao Huang, Danfang Zhang

**Affiliations:** 1 Department of Pathology, Tianjin Medical University, Tianjin 300070 Tianjin, China; 2 Tianjin Medical University Cancer Institute and Hospital, National Clinical Research Center for Cancer, Key Laboratory of Cancer Prevention and Therapy, Tianjin’s Clinical Research Center for Cancer, 300060 Tianjin, China

**Keywords:** Tumour angiogenesis, Necroptosis

## Abstract

Necroptosis is a caspase-independent form of programmed cell death. Receptor interacting protein kinase 1 (RIPK1) is a key molecule in the initiation of necroptosis and the formation of the necrotic complex. Vasculogenic mimicry (VM) provides a blood supply to tumor cells that is not dependent on endothelial cells. However, the relationship between necroptosis and VM in triple-negative breast cancer (TNBC) is not fully understood. In this study, we found that RIPK1-dependent necroptosis promoted VM formation in TNBC. Knockdown of RIPK1 significantly suppressed the number of necroptotic cells and VM formation. Moreover, RIPK1 activated the p-AKT/eIF4E signaling pathway during necroptosis in TNBC. eIF4E was blocked by knockdown of RIPK1 or AKT inhibitors. Furthermore, we found that eIF4E promoted VM formation by promoting epithelial-mesenchymal transition (EMT) and the expression and activity of MMP2. In addition to its critical role in necroptosis-mediated VM, eIF4E was essential for VM formation. Knockdown of eIF4E significantly suppressed VM formation during necroptosis. Finally, through clinical significance, the results found that eIF4E expression in TNBC was positively correlated with the mesenchymal marker vimentin, the VM marker MMP2, and the necroptosis markers MLKL and AKT. In conclusion, RIPK1-dependent necroptosis promotes VM formation in TNBC. Necroptosis promotes VM formation by activating RIPK1/p-AKT/eIF4E signaling in TNBC. eIF4E promotes EMT and MMP2 expression and activity, leading to VM formation. Our study provides a rationale for necroptosis-mediated VM and also providing a potential therapeutic target for TNBC.

## Introduction

Breast cancer (BC) is one of the most common malignancies among women worldwide, and its incidence is increasing every year [1]. The latest statistics for 2020 show 2.26 million new BC patients worldwide, making it the number one cancer [2]. Among the different molecular types of BC, triple-negative (estrogen receptor (ER), progesterone receptor (PR) and HER2-negative) subtypes of breast cancer (TNBC) have a high risk of metastasis and recurrence. TNBC has a poor prognosis, as ER-, PR- or HER2- targeted therapies are not effective against this subtype, and other treatments are limited [3, 4].

In the hypoxic microenvironment of solid tumors, tumor cells and extracellular matrix remodel to form tubular structures that mimic the function of blood vessels and supply nutrients and oxygen for tumor growth and invasion, and such tubular structures have been termed as vasculogenesis mimicry (VM) [5]. The three key components of vascular mimetic induction include self-deformation of the malignant tumor cells, remodeling of the tumor cells with the extracellular matrix and connection of the VM to the host-derived vasculature [6]. Epithelial-mesenchymal transition (EMT) is a process by which epithelial cells acquire a mesenchymal cell phenotype and lose their intercellular and extracellular matrix adhesions, allowing them to dissociate from the primary tumor [7]. The occurrence of EMT in tumor cells has been suggested to be crucial in the formation of VM [8]. The effect of tumor vascular abnormalities on the surrounding tumor microenvironment can lead to increased hypoxia and increased intrastromal pressure, which in turn can lead to treatment resistance [9]. Angiomimetic states are observed in BC patients, and treatment resistance occurs when these patients are treated with antiangiogenic therapy [10]. TNBC has a strong proliferative activity, and a large number of new blood vessels provide nutrients for tumor growth, promoting its development and invasion and metastasis [11]. Therefore, targeted angiogenic and antiangiogenic therapy is an effective way to control TNBC progression and treat tumors.

Necroptosis is a caspase-independent form of programmed cell death that begins with the activation of receptor interacting protein kinase 1(RIPK1) and RIPK3 and the formation of the necrosome [12, 13]. Mixed-lineage kinase domain-like protein (MLKL) is a substrate for RIPK3 kinase, which phosphorylates its substrate MLKL, followed by its translocation to the plasma membrane to participate in necroptosis, leading to increased plasma membrane permeability and disruption of integrity, ultimately leading to cell death [12, 14]. Necrostatin-1(Nec-1) inhibits RIPK1 kinase activity, blocking the RIPK1/RIPK3 interaction and necroptosis [15].

Eukaryotic translation initiation factor 4E (eIF4E) recognizes and binds mRNA caps containing 7-methylguanosine at an early stage of protein synthesis and facilitates ribosome binding by inducing the release of mRNA secondary structures [16]. eIF4E expression and activation are closely associated with cell transformation and tumorigenesis [17]. The activity of eIF4E is regulated by PI3K (phosphatidylinositol 3-kinase)/AKT (also known as protein kinase B, PKB)/mTOR (target of rapamycin) [18]. It has been shown that in TNBC, patients with high eIF4E expression are more likely to relapse and have a higher mortality rate than those with low eIF4E expression [19]. RIPK1 promotes the transcription of inflammatory cytokines by activating the eIF4E-driven cap-dependent translation initiation pathway [19].

According to previous studies, necroptosis of tumor cells can both promote tumor cell metastasis and provide growth space for surviving cells [12, 20]. We hypothesized that RIPK1-dependent necroptosis in TNBC promotes VM formation and explored the role of eIF4E in this process.

## Materials and methods

### Cell culture

The human breast cancer cell lines MDA-MB-231, Hs578T, and MCF-7 were purchased from Shanghai FuHeng Biotechnology Co. Ltd. HUVECs were obtained from ATCC in 2012. All cells used in the experiments were cultured in DMEM supplemented with 10% fetal bovine serum (Invitrogen, CA, USA) and 1% penicillin/streptomycin in an incubator at 37 °C with 5% CO_2_. All the cell lines were recently authenticated by PCR-STR analysis.

### Stable transfection using lentiviral infection

We used HEK293T cells for lentivirus production, purification, and infection according to the manufacturer’s instructions (Lenti-Pac^TM^ HIV Expression Kit, Genecopoeia). The plasmids containing RIPK1 complementary cDNA (EX-O0078-Lv122), eIF4E complementary cDNA (EX-A4264-Lv122), a negative control (EX-NEG-Lv122), RIPK1 small interfering RNAs (HSH114959-LVRU6 GP), and a SH-Control (CSHCTR001-LVRU6 GP), were synthesized by Genecopoeia. The PLKO.1-puro vector was used to clone the SH-RNAs targeting eIF4E. The sequence of SH-eIF4E was 5′-CCGGCCAAAGATAGTGATTGGTTATCTCGAGATAACCA ATCACTATCTTTGGTTTTTG-3′. The plasmids were transfected into HEK293T cells, and the supernatant containing the virus was collected at 48 h. The virus was then concentrated and used in combination with polybrene to infect breast cancer cells. The infected cells were selected with puromycin for at least 1 week to obtain stable control cell lines; cells infected with empty lentiviral vectors were also set up.

### Necroptosis induction and staining

Cells were treated with z-VAD-fmk (20 µM) and AZD5528 (10 µM) for 30 min, followed by TNF-α (30 ng/ml) treatment for 3–4 h (TZA treatment). To test the effects of necrostatin-1 (30 µM; for 1 h), GSK’872 (10 µM; for 2 h), NSA (1.5 µM; for 12 h), and the AKT inhibitor MK-2206 (0–8 µM; for 12 h), cells were pretreated with each of the reagents at the indicated concentrations and time before the addition of TNF-α (30 ng/ml)/ z-VAD-fmk (20 µM)/ and AZD5528 (10 µM). After induction, PI^+^ staining media (Beyotime) was added and incubated with the cells for 20 min. Images were acquired by fluorescence microscopy(Nikon), and the proportion of necroptotic cells in each group of cells was observed.

### Conditioned medium collection

After treatment with TZA for the indicated times, the medium was replaced with fresh medium, and incubation was continued for 12 h. Then, the culture medium was collected. The medium was filtered through a 0.22 μm filter and centrifuged at 1500 rpm for 5 min, and the supernatant was collected.

### 3D Matrigel culture

The bottom of a 24-well plate was coated with 30 μl Matrigel (BD, USA) diluted 1:3 with DMEM. The 24-well plate was irradiated and dried on an ultraclean bench and then placed in a 37 °C cell incubator for hydration. Tumor cells were suspended in culture medium or conditioned medium, added to the 24-well plate and incubated for 24–36 h at 37 °C. An inverted microscope captured the number of VM tubes. Each condition was assessed with at least three independent experiments.

### Western blotting analysis

Protein was extracted using SDS lysis buffer and transferred to PVDF membranes. After the membranes were blocked with 5% skim milk for 1 h, they were incubated with primary antibodies overnight at 4 °C, which was followed by incubation with secondary antibodies for 2 hours. Bands were visualized using a C-Digit Blot Scanner (Gene Company) and analyzed with ImageJ software. GADPH (1:1000, sc-47724, Santa Cruz) was used as a protein loading control. The following primary antibodies were used: antibodies against eIF4E (1:200, sc-9976) from Santa Cruz, RIPK1 (1:500, ab106393), VE-cadherin (1:500, ab33168), vimentin (1:1000, ab92547), and Snail (1:1000, ab180714) from Abcam (Cambridge, USA), phospho-AKT (S473) (1:500, T40067F) from Abmart, AKT (1:500, #AF6259) from Affinity, E-cadherin (1:1000, #3195) from Cell Signaling Technology, and MMP-2 (1:500, 10373-2-AP) from Proteintech.

### Immunofluorescence staining

The cells were plated on coverslips, incubated at 37 °C overnight, permeabilized with 0.1% Triton X-100 and blocked with 5% FBS. Then, the cells were incubated with primary antibodies against eIF4E (sc-9976; Santa Cruz; 1:100), E-cadherin (#3195; Cell Signaling Technology; 1:100), and vimentin (ab92547; Abcam; 1:100). After incubation with fluorophore-conjugated secondary antibodies, the nuclei were counterstained with DAPI (Sigma). Images were acquired by fluorescence microscopy.

### Wound-healing assay

Cells were seeded in 24-well plates. When the cells reached confluency, a wound was created using a 100 μL sterile pipette tip and then photographed (0 hour). The rate of gap closure was measured at different time points. Each experiment was performed three times.

### Cell invasion and cell migration assays

Migration assays were performed with breast cancer cells (1 × 10^5^) that were added to the upper chamber with serum-free medium, and DMEM with 10% FBS was added to the bottom chamber in 24-well plates. After incubation for 24 h, the cells were fixed with methanol and stained with crystal violet for 20 minutes. Invasion assays were performed similarly to the migration assays except that the transwell chambers were coated with Matrigel before the cells were seeded in the upper chamber. These cells were counted using an inverted light microscope (Nikon). Each experiment was performed three times.

### Gelatin zymography assays

To assess MMP-2 activity in MDA-MB-231 and MCF7 cells, including the control and treatment groups, we collected serum-free conditioned medium for SDS‒PAGE using a 10% polyacrylamide gel containing 0.01% gelatin. The gels were loaded with 30 μl of medium from each sample, and electrophoresis was conducted in ice water at 120 V for 3 h. After electrophoresis, the gels were equilibrated 4 times in 2.5% Triton X-100 for 30 min. Subsequently, the gels were incubated in substrate buffer (1 M Tris-HCl pH 7.5, 0.1 M CaCl_2_ and 100 mM ZnCl_2_) for 42 h at 37 °C and then stained with Coomassie Brilliant Blue R250 for 2 h with gentle shaking. The gels were subsequently washed until clear bands appeared.

### Real‐time PCR

Total RNA extracted from MDA-MB-231 and MCF7 cells, including the control and treatment groups, was isolated with TRIzol‐A^+^ Reagent (TIANGEN, Beijing, China), and cDNA was synthesized using the PrimeScript™ RT Reagent Kit with gDNA Eraser (TaKaRa, Dalian, China). The following real‐time PCR primers were used: Rictor (forward: 5′‐TTTCGGGGATTTCTGGATG‐3′, reverse: 5′‐AAAGCCCAGTC TCATGACATT‐3′), MMP‐2 (forward: 5′‐AAGGATGGCAAGTACGGCTT‐3′, reverse: 5′‐CGCTGGTACAGCTCTCATACTT‐3′) and GAPDH (forward: 5′‐GGA GCGAGATCCCTCCAAAAT‐3′, reverse: 5′‐GGCTGTTGTCATACTTCTCATGG‐3′). PCR was performed using real‐time PCR Master Mix (SYBR Green) according to the manufacturer’s instructions. Signals were detected with an ABI 7500 Real‐Time PCR System (Applied Biosystems, California, USA).

### Xenograft animal models

The methods for the animal experiments were approved by the Tianjin Medical University Ethics Committee. All procedures were carefully conducted to protect the welfare of the animals and prevent their suffering. Tientsin Albino 2 (TA2) female mice (4–6 weeks, *n* = 24), which are spontaneous breast cancers models, were provided by the Animal Center of Tianjin Medical University. All animal experiments have complied with the ARRIVE guidelines. No animals suffered unnecessarily hurt at any stage of an experiment. We collected the breast tumors, and the cancer cells (5 × 10^6^ cells) were injected subcutaneously into theroin region of the TA2 mice. Mice carrying tumors were randomly divided into four groups. When the tumors reached 0.15 cm^3^, SH-eIF4E plasmids (*n* = 6, 6.25 µg) and control plasmids(*n* = 6, 6.25 µg) along with the RNA transfection reagent (Entransterin vivo, Engreen) were injected into the tumor every 2 days. Similarly, SH-RIPK1 plasmids (*n* = 6, 6.25 µg) and control plasmids(*n* = 6, 6.25 µg) along with the RNA transfection reagent were injected into the tumor every 2 days. We measured the change in tumor volume (length × width^2^/2). These TA2 mice were simultaneously sacrificed after 5 injections.

### Immunohistochemistry staining

The tissues were deparaffinized in xylene and rehydrated in graded alcohols. First, 3% H_2_O_2_ was used to block endogenous peroxidase, followed by microwave retrieval. Tissue sections were blocked in 10% goat serum (Zhongshan Chemical Co., Beijing, China) and incubated consecutively with primary antibodies and a secondary antibody. The following primary antibodies were used: antibodies against eIF4E (1:50, sc-9976) from Santa Cruz, RIPK1 (1:50, Proteintech), VE-cadherin (1:100, ab33168), vimentin (1:200, ab92547), phospho-AKT (S473) (1:50, T40067F) from Abmart, E-cadherin (1:200, #3195) from Cell Signaling Technology, MMP-2 (1:250, 10373-2-AP) from Proteintech and MLKL(1:100, sc-293201) from Santa Cruz. DAB staining was performed for appropriate durations, and all sections were counterstained with hematoxylin. PBS was used in place of the primary antibodies for all negative controls. The staining intensity was scored as follows: 0 (negative), 1 (weak), 2 (medium) and 3 (high). The percentage of stained cells in the whole field was scored as follows: 0 (negative), 1 (≤25%), 2 (25–50%), and 3 (>50%). The final score was determined by adding the scores of the staining intensity and percentage of stained cells together.

### Statistical analysis

The experimental data and images were analyzed and plotted using GraphPad Prism 5. The means between two groups were compared by Student’s *t* test, and the means between multiple groups were compared by ANOVA. *P* < 0.05 was considered to be a significant difference.

## Results

### RIPK1-dependent necroptosis promotes VM formation in TNBC

We first examined the background expression of RIPK1 by Western blotting in the human BC cell lines MDA-MB-231 and MCF-7. As shown in Fig. S1A, B, the expression level of RIPK1 was significantly higher in MDA-MB-231 cells than in MCF-7 cells (*t* = 8.48, *P* < 0.05). Next, we constructed a stably transfected cell line by downregulating RIPK1(SH-RIPK1) in MDA-MB-231 cells and upregulating RIPK1(EX-RIPK1) in MCF-7 cells by lentiviral transfection (Fig. S1C, D).

Next, we used the TNBC cell lines MDA-MB-231 and Hs578T to detect the number of necroptotic cells after TNFα, TNFα/Z-VAD (TZ), Z-VAD/AZD5528 (ZA), and TNFα/Z-VAD/AZD5528 (TZA) stimulation. As shown in Fig. S2A, the number of necroptotic MDA-MB-231 and Hs578T cells increased significantly after treatment with TZ and TZA, suggesting that TZ and TZA (but not TNFα alone or ZA) could activate necroptosis in TNBC cells. TNFα can activate apoptosis and NF-κB signaling. Z-VAD is a pancaspase inhibitor. AZD5528 is an IAP antagonist that promotes the deubiquitination and release of RIPK1 from the TNFR1 complex. AZD5528 leads to a switch from survival to death signaling complexes [21]. Thus, our results show that TNFα/Z-VAD, and TNFα/Z-VAD/AZD5528 can effectively induce necroptosis in TNBC cells.

**Fig. 1:**
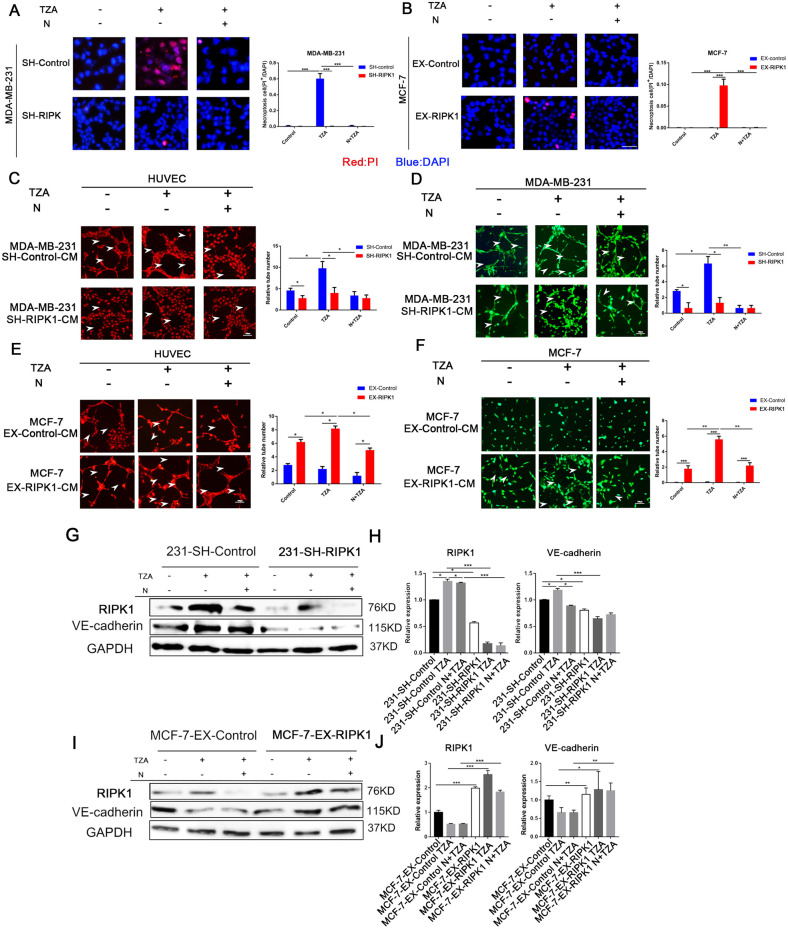
RIPK1-dependent necroptosis promotes VM formation in triple-negative breast cancer. **A**, **B** RIPK1 expression promotes necroptosis. RIPK1-knockdown and negative control shRNA MDA-MB-231 cells were treated with TNF (30 ng/ml), z-VAD-fmk (20 µM), and AZD5528 (TZA) in the absence or presence of necrostatin-1 (30 µM; N + TZA) for 4 h, and cell death was determined by fluorescence microscopy. RIPK1 overexpressing and negative control MCF-7 cells were treated with the same method. Red: PI (propidium iodide), blue: DAPI. (***P < 0.001, bar = 200 μm). **C**, **D** Necroptotic MDA-MB-231 cell-conditioned medium promoted three-dimensional tube formation in HUVECs (**C**) and MDA-MB-231 cells (**D**). MDA-MB-231 and HUVECs were treated with SH-RIPK1 and control conditioned medium (control, TZA, N + TZA) for 3D Matrigel culture, and the number of tubes was counted. **E**, **F** MCF-7 EX-RIPK1-conditioned medium promoted HUVEC (**E**) and MCF-7 (**F**) three-dimensional tube formation. MCF-7 and HUVECs were treated with EX-RIPK1 and control conditioned medium (control, TZA, N + TZA) for 3D Matrigel culture, and the number of tubes was counted. (*P < 0.05, ***P < 0.001, bar = 100 μm). **G**–**J** Necroptosis and RIPK1 expression promoted the expression of the VM marker VE-cadherin in MDA-MB-231 (**G**, **H**) and MCF-7 (**I**, **J**) cells, as shown by Western blotting and quantitative analysis. SH-RIPK1 and control MDA-MB-231 cells were treated with TZA or N + TZA. EX-RIPK1 and control MCF-7 cells were treated with the same method. Data are representative of three independent experiments. (one-way ANOVA).

To further investigate the role of RIPK1 in necroptosis, we established conditions to induce necroptosis in BC cells using TNFα/Z-VAD/AZD5528 administration. Staining for necroptosis showed that the number of necroptotic cells was significantly higher in the TNBC cell line MDA-MB-231 that was treated with TZA (*P* < 0.001; Fig. 1A). In contrast, there was no significant increase in the number of necroptotic MCF-7 cells treated with TZA (Fig. 1B). In MDA-MB-231 cells, the number of necroptotic cells was significantly inhibited after treatment with Nec-1, and the number of necroptotic cells was significantly lower among MDA-MB-231 SH-RIPK1 cells treated with TZA than in control cells treated with TZA (*P* < 0.001; Fig. 1A). The number of necroptotic cells was significantly higher among MCF-7 EX-RIPK1 cells treated with TZA (*P* < 0.001) (Fig. 1B).

We collected necroptotic conditioned medium with or without Nec-1 to explore its effect on tube-forming capacity in HUVECs. The results in Fig. 1C show that the number of tubes was significantly increased in HUVECs treated with TZA-conditioned medium compared with the control (*P* < 0.05). Conditioned medium containing Nec-1 significantly inhibited tube formation compared with TZA-conditioned medium (*P* < 0.05), and the number of tubes was significantly reduced after treatment with MDA-MB-231 SH-RIPK1-conditioned medium compared with the control (*P* < 0.05). There was no significant difference in MCF-7 cells treated with TZA compared with the control. The number of tubes was increased in HUVECs treated with EX-RIPK1 conditioned medium and EX-RIPK1-TZA conditioned medium (*P* < 0.05), but tube formation was inhibited after treatment with Nec-1 (Fig. 1E). Similarly, tube-formation capacity in breast cancer cells was observed after treatment with conditioned medium. The results showed that the number of tubes was significantly increased in MDA-MB-231 cells treated with TZA-conditioned medium compared with the control (*P* < 0.05), but conditioned medium containing Nec-1 or MDA-MB-231 SH-RIPK1-conditioned medium significantly inhibited tube formation compared with TZA-conditioned medium. (*P* < 0.05). The number of tubes was increased in the MCF-7 cells treated with MCF-7 EX-RIPK1 conditioned medium and EX-RIPK1-TZA conditioned medium (*P* < 0.001), as shown in Fig. 1D, F. We use cell-conditioned medium treated with TZA to incubate HUVEC and Hs578T. The results show that increased the number of HUVEC and Hs578T cell tubes formed, and pretreatment of Hs578T cell-conditioned medium with Nec-1 blocked TZA-induced HUVEC and Hs578T cell tube formation (*P* < 0.001; Fig. S2B).

The expression levels of the VM marker VE-cadherin following necroptosis were further analyzed by Western blotting. Fig. 1G, H shows that VE-cadherin expression levels were significantly increased after treatment with TZA in MDA-MB-231 cells (*P* < 0.05) but were significantly decreased (*P* < 0.05) after treatment with Nec-1 or RIPK1 knockdown. MCF-7 EX-RIPK1 significantly increased VE-cadherin expression levels (*P* < 0.05), and the results were consistent with the results of VM formation in tumor cells (Fig. 1I, J). Western blotting was used to detect RIPK1 and VE-cadherin expression after T, TZ, ZA, and TZA stimulation in MDA-MB-231 cells. We found that TZA or TZ (but not T alone or ZA) significantly increased RIPK1 and VE-cadherin levels compared with the control. This suggests that during TZA- or TZ-induced necroptosis, VE-cadherin expression is activated (Fig. S3A).

### RIPK1-dependent necroptosis in TNBC regulates eIF4E expression via p-AKT

**Fig. 2:**
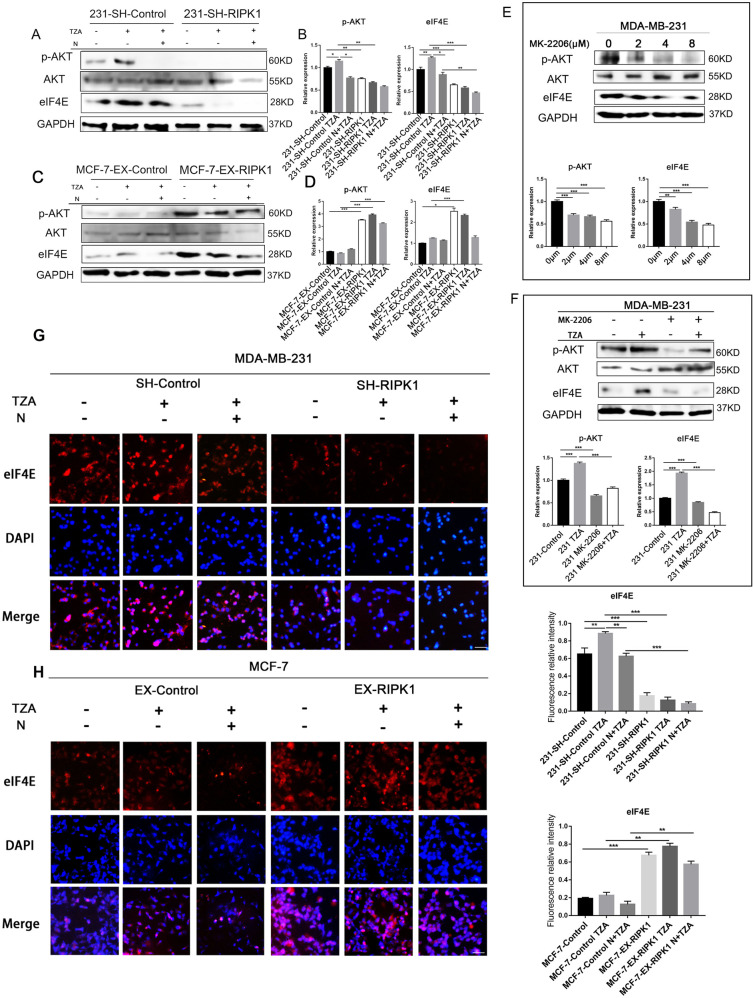
RIPK1-dependent necroptosis in triple-negative breast cancer regulates eIF4E expression via p-AKT. **A**–**D** Necroptosis and RIPK1 expression promote the expression of p-AKT and eIF4E in MDA-MB-231 (**A**, **B**) and MCF-7 (**C**, **D**) cells, as shown by Western blotting and quantitative analysis. SH-RIPK1 and control MDA-MB-231 cells were treated with TZA or N + TZA. EX-RIPK1 control MCF-7 cells were treated with the same method. **E** The p-AKT inhibitor MK-2206 inhibited eIF4E expression in MDA-MB-231 cells, as shown by Western blotting and quantitative analysis. MDA-MB-231 cells were treated with MK-2206 at the indicated concentrations (0μM, 2μM, 4μM and 8μM). **F** MK-2206 (8 μM) blocked the expression of eIF4E in necroptosis, as shown by Western blotting and quantitative analysis. **G**, **H** Immunofluorescence staining results showed the effect of necroptosis and RIPK1 expression levels on the expression and localization of eIF4E in MDA-MB-231 (**G**) and MCF-7 (**H**) cells. Data are representative of three independent experiments (*P < 0.05, **P < 0.01, ***P < 0.001, bar = 200 μm). (one-way ANOVA).

To investigate whether RIPK1 affects eIF4E expression through p-AKT in BC, we examined the effect of necroptosis and RIPK1 expression on p-AKT levels by Western blotting. Fig. 2A, B shows that eIF4E expression was increased significantly after treatment with TZA in MDA-MB-231 cells but not in RIPK1- knockdown MDA-MB-231 cells. eIF4E expression was significantly decreased after pretreatment with Nec-1. eIF4E expression was significantly higher in MCF-7 EX-RIPK1 cells treated with TZA(*P* < 0.05) (Fig. 2C, D). In addition, the same trend in p-AKT expression levels was observed. p-AKT levels were significantly increased after treatment with TZA in MDA-MB-231 cells but not in RIPK1 knockdown MDA-MB-231 cells and were significantly decreased after pretreatment with Nec-1 (*P* < 0.05). Total AKT expression was not affected (Fig. 2A, B). Overexpression of RIPK1 significantly promoted p-AKT expression in MCF-7 cells (*P* < 0.05; Fig. 2C, D). This result is consistent with the changes in eIF4E expression. This suggests that p-AKT and eIF4E are positively correlated in RIPK1-dependent necroptosis in TNBC.

Wester blotting was used to detect p-AKT/eIF4E levels after T, TZ, ZA and TZA stimulation in MDA-MB-231 cells. We found that TZA or TZ (but not T alone or ZA) significantly increased p-Akt and eIF4E levels compared with the control. This suggests that during TZA- or TZ-induced necroptosis, p-AKT/eIF4E signals are activated. p-AKT and eIF4E were not induced by TNFα alone or ZA but required specific necroptotic signaling by TNF-α/z-VAD-fmk/AZD5528 or TNF-α/z-VAD-fmk together (Fig. S3A). The specific inhibitors GSK’872 and NSA are widely used in various experimental models to target the kinase activities of RIPK3 and MLKL, respectively, to elucidate the roles of necroptosis components [22–24]. Western blot experiments showed that in the TNBC cell lines MDA-MB-231 and Hs578T, GSK’872 treatment reversed the increase in p-AKT and eIF4E in response to TZA stimulation. However, NSA treatment could not reverse this increase. Thus, during necroptosis, RIPK1 can activate p-AKT/eIF4E, but MLKL cannot. Interestingly, RIPK3 inhibitors blocked p-AKT/eIF4E in response to TZA stimulation, possibly due to RIPK3 inhibitors blocking RIPK1-RIPK3 necrosis complex formation. (Fig. S3B)

To further investigate the regulation of eIF4E by p-AKT, MDA-MB-231 cells were treated with an AKT inhibitor (MK-2206) at the indicated concentrations. Figure 2E shows that eIF4E expression was subsequently inhibited with increasing concentrations of AKT inhibitors (*P* < 0.05). eIF4E levels were significantly increased in MDA-MB-231 cells treated with TZA (Fig. 2F), but MK-2206 (8 μM) pretreatment inhibited the increase in eIF4E (*P* < 0.001). It was confirmed that during necroptosis, the phosphorylation and activation of AKT downstream substrates in response to TZA is RIPK1-dependent. RIPK1/p-AKT elevates eIF4E expression during necroptosis.

We further verified the effect of necroptosis and RIPK1 on the expression and localization of eIF4E by immunofluorescence staining (Fig. 2G). The results showed that eIF4E expression was increased in the nucleus and plasma in MDA-MB-231 cells treated with TZA. But eIF4E expression was not increased in RIPK1-knockdown MDA-MB-231 cells, and eIF4E expression decreased after pretreatment with Nec-1. In MCF-7 EX-RIPK1 cells treated with TZA, eIF4E expression was increased in the nucleus and plasma. This result suggested that necroptosis increases eIF4E expression in a RIPK1-dependent manner in TNBC.

### eIF4E promotes RIPK1-dependent necroptosis-mediated VM formation in TNBC

We first examined the background expression of eIF4E by Western blotting in the human BC cell lines MDA-MB-231 and MCF-7. As shown in Fig. S4A, B, the expression level of eIF4E was significantly higher in MDA-MB-231 cells than in MCF-7 cells (*t* = 25.84, *P* < 0.01). Next, we constructed stably transfected cell line by downregulating eIF4E in MDA-MB-231 cells and upregulating eIF4E in MCF-7 cells by lentiviral transfection (Fig. S4C, D).

**Fig. 3:**
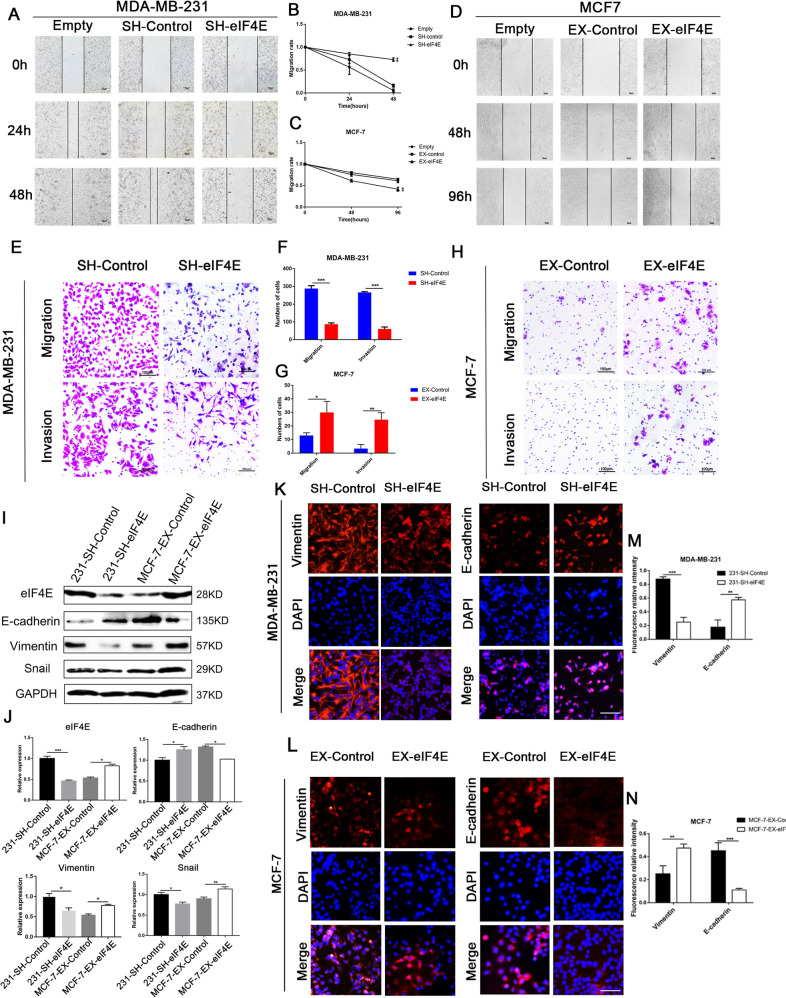
eIF4E expression promotes migration, invasion and EMT. **A**–**D** The wound-healing assay was conducted on SH-eIF4E MDA-MB-231, EX-eIF4E MCF-7, and control cells. **E**–**H** Migration and invasion assays were conducted on SH-eIF4E MDA-MB-231, EX-eIF4E MCF-7, and control cells. **I**, **J** The protein levels of EMT-related markers in MDA-MB-231-SH-eIF4E, MCF-7-EX-eIF4E and control cells were determined by Western blotting. (*P < 0.05, **P < 0.01, ***P < 0.001, bar = 100 μm). **K**, **L** The immunofluorescence assay results showed that eIF4E promoted the expression of EMT marker proteins (bar = 200 μm). **M**, **N** The quantitative analysis of the eIF4E-promoting EMT immunofluorescence. (**P < 0.01, ***P < 0.001) Data are representative of three independent experiments (Student’s *t* test).

First, we examined the effect of eIF4E on the healing ability of BC cells by scratch assays. The results showed that downregulation of eIF4E inhibited the migration of MDA-MB-231 cells and that overexpression of eIF4E promoted the migration of MCF-7 cells (*P* < 0.01) (Fig. 3A–D). Furthermore, the migration assay results are shown in Fig. 3E–H, and SH-eIF4E MDA-MB-231 cells had a significantly lower migratory capacity than the control cells (*t* = 8.8, *P* < 0.001). EX-eIF4E significantly promoted the migratory capacity of MCF-7 cells compared to MCF-7 control cells (*t* = 4.7, *P* < 0.05). Similarly, consistent results were observed in the Matrigel invasion assay (*P* < 0.01). These results reveal that eIF4E promotes the migration and invasive ability of BC in vitro.

We examined the effect of eIF4E on the protein expression of EMT markers by Western blotting. The results showed that in MDA-MB-231 cells, the expression level of the epithelial marker E-cadherin was increased and the expression level of the mesenchymal marker vimentin and the transcription factor Snail was decreased in SH-eIF4E cells compared with the control (*P* < 0.05); in MCF-7 cells, compared with the control, the expression level of E-cadherin in EX-eIF4E cells was decreased, and vimentin and Snail expression levels were increased (*P* < 0.05; Fig. 3I, J). We further validated this result by immunofluorescence staining and examined the cellular localization and expression of marker molecules (Fig. 3K–N), which showed that eIF4E expression promoted the expression of EMT markers in BC (*P* < 0.05).

**Fig. 4:**
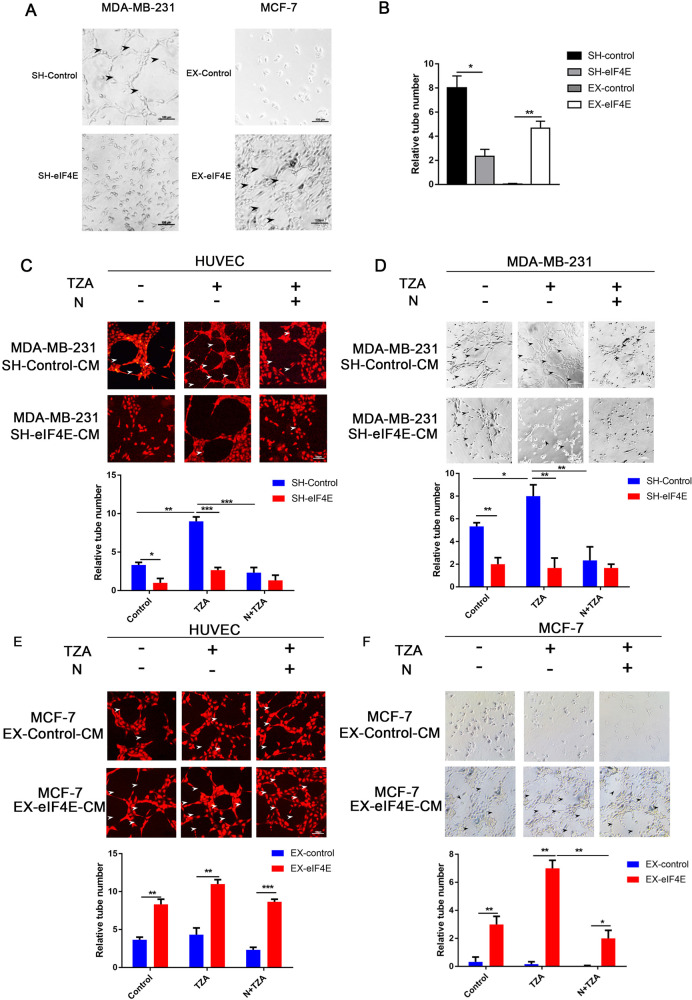
eIF4E promotes RIPK1-dependent necroptosis-mediated VM formation in triple-negative breast cancer. **A**, **B** Three-dimensional tube formation showing that eIF4E expression promoted VM formation. SH-eIF4E MDA-MB-231, EX-eIF4E MCF-7, and control cells for 3D Matrigel culture (bar = 100 μm). **C**, **D** Conditioned medium from SH-eIF4E MDA-MB-231 cells with induced necroptosis reduced the number of 3D tubes formed in HUVECs and MDA-MB-231 cells. MDA-MB-231 and HUVECs were treated with SH-eIF4E and control conditioned medium (control, TZA, N + TZA) for 3D Matrigel culture, and the number of tubes was counted. **E**, **F** Conditioned medium from EX-eIF4E MCF-7 cells with induced necroptosis increased the number of 3D tube formations in HUVECs and MCF-7 cells. MCF-7 and HUVECs were treated with EX-eIF4E and control conditioned medium (control, TZA, N + TZA) for 3D Matrigel culture, and the number of tubes was counted (bar = 100 μm). Data are representative of three independent experiments (*P < 0.05, **P < 0.01, ***P < 0.001) (one-way ANOVA).

To investigate the effect of eIF4E on VM formation, we first explored the effect of changes in eIF4E expression levels on the tube-forming ability of BC cells using three-dimensional culture. As shown in Fig. 4A, B, the number of ducts formed by MDA-MB-231 SH-eIF4E cells was significantly reduced compared to that formed by the control cells (*t* = 8.5, *P* < 0.05); duct formation was observed in MCF-7 EX-eIF4E cells, but MCF-7 cells did not form ducts (*t* = 13.83, *P* < 0.05). These results suggest that eIF4E expression can promote VM formation in BC cells.

We further explored the role of eIF4E in necroptosis-mediated VM formation in TNBC. The results in Fig. 4C, D show that the number of tubes formed was significantly increased in HUVECs and MDA-MB-231 cells treated with TZA-conditioned medium compared with the control (*P* < 0.01), but conditioned media containing Nec-1 or MDA-MB-231 SH-eIF4E-conditioned media significantly inhibited tube formation compared with TZA-conditioned media (*P* < 0.05). There was no significant difference in MCF-7 control cells treated with TZA compared with the control. The number of tubes was increased in HUVECs and MCF-7 cells treated with EX-eIF4E conditioned media and EX-eIF4E TZA conditioned media (*P* < 0.05), but tube formation in MCF-7 cells was inhibited after treatment with Nec-1 (Fig. 4E, F). These results suggest that eIF4E promotes necroptosis-mediated VM formation in TNBC.

### eIF4E promotes VM formation in TNBC by promoting MMP2 expression and enzymatic activity

**Fig. 5:**
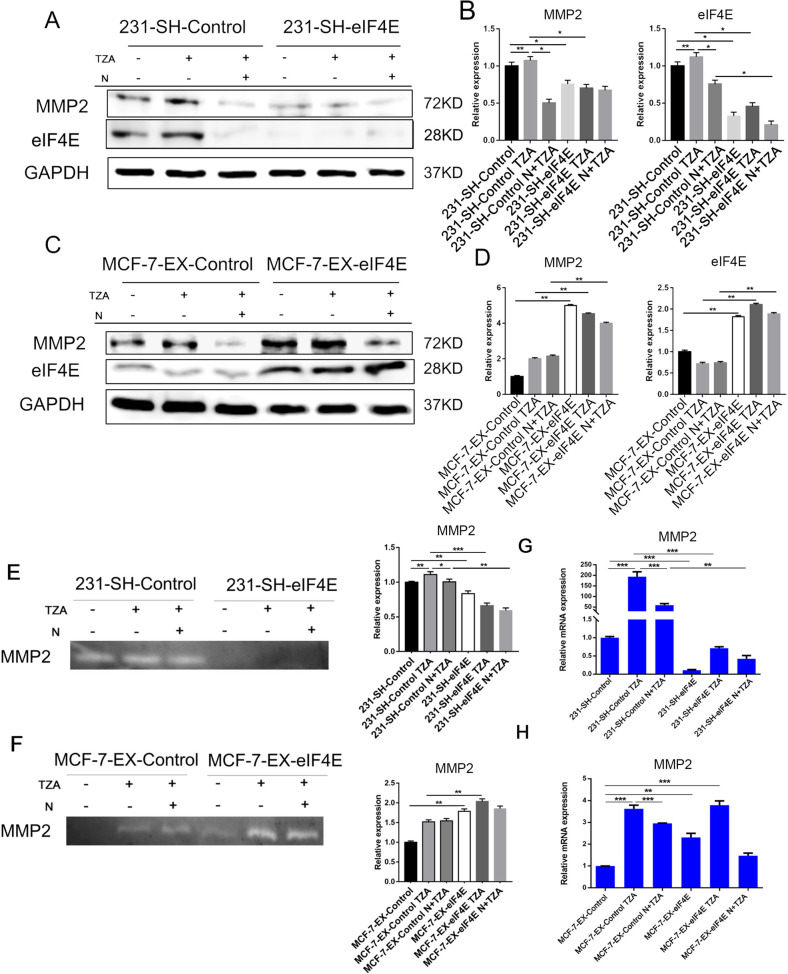
eIF4E promotes MMP2 expression and enzymatic activity in necroptosis. **A**–**D** eIF4E promoted the protein expression of MMP2 during necroptosis, as shown by Western blotting and quantitative analysis. **E**, **F** eIF4E promoted MMP2 enzymatic activity in necroptosis, as shown by gelatin zymography. **G**, **H** eIF4E promoted the RNA expression of MMP2 in necroptosis by RT-PCR. SH-eIF4E and control MDA-MB-231 cells were treated with TZA or N + TZA. EX-eIF4E and control MCF-7 cells were treated with the same method. Data are representative of three independent experiments (*P < 0.05, **P < 0.01, ***P < 0.001) (one-way ANOVA).

The Western blot results showed that necroptosis in MDA-MB-231 cells promoted MMP2 expression (*P* < 0.01), and Nec-1 significantly inhibited MMP2 expression (*P* < 0.05). eIF4E knockdown significantly inhibited MMP2 expression induced by TZA (*P* < 0.05; Fig. 5A, B). Overexpression of eIF4E in MCF-7 cells could significantly promote MMP2 expression levels (*P* < 0.01); in MCF-7 control cells, there was no significant change after treatment with TZA (Fig. 5C, D). Similarly, the gelatin zymography results showed that necroptosis promoted MMP2 activity in MDA-MB-231 cells, which was inhibited by Nec-1 (*P* < 0.01), and eIF4E knockdown significantly inhibited MMP2 enzyme activity (*P* < 0.01; Fig. 5E). Overexpression of eIF4E in MCF-7 cells could significantly promote MMP2 enzyme activity, and this activity was enhanced after treatment with TZA in EX-eIF4E cells (*P* < 0.01; Fig. 5F). Subsequently, the results were further validated by RT‒PCR. Fig. 5G shows that necroptosis in MDA-MB-231 cells promoted MMP2 RNA levels and Nec-1 pretreatment significantly inhibited MMP2 levels (*P* < 0.001). eIF4E knockdown significantly inhibited MMP2 RNA levels induced by TZA (*P* < 0.001). In MCF-7 cells, MMP2 RNA levels were elevated after treatment with TZA (*P* < 0.001), but MMP2 RNA levels were inhibited by Nec-1 pretreatment (*P* < 0.001). eIF4E overexpression could significantly promote MMP2 RNA levels (*P* < 0.05; Fig. 5H).

### eIF4E promotes BC growth and the expression of EMT markers and MMP2 in the TA2 mouse model

**Fig. 6:**
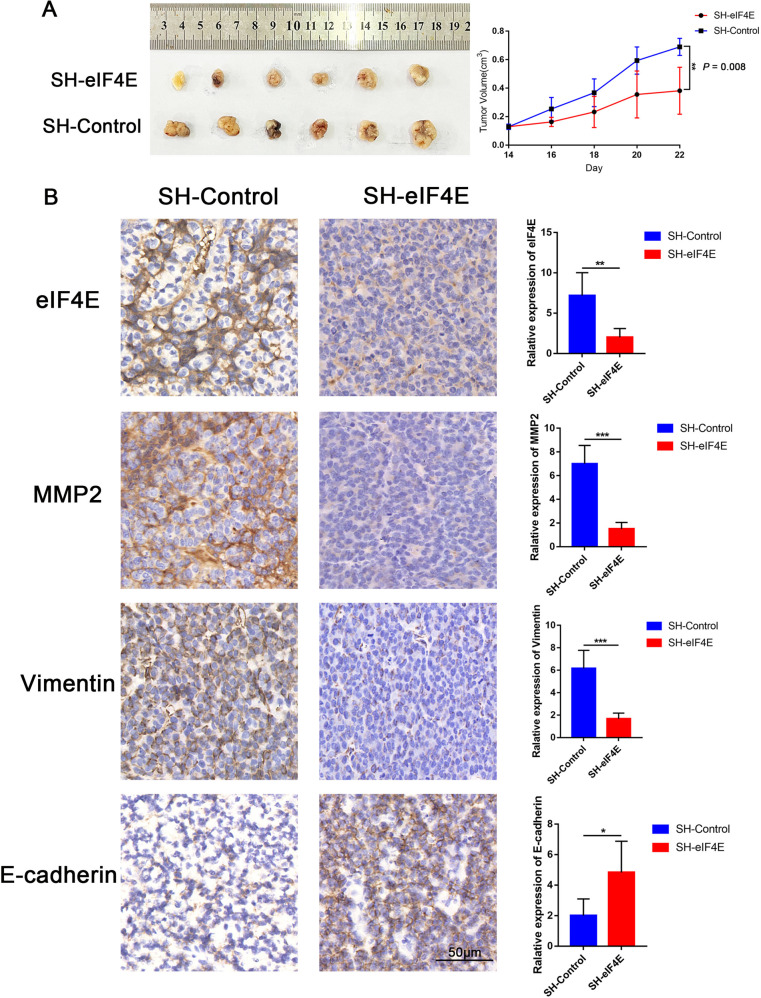
eIF4E promotes breast cancer growth and the expression of EMT markers and MMP2 in the TA2 mouse model. **A** Tumor growth curves showed that SH-eIF4E inhibited tumor growth. **B** The expression levels of eIF4E, MMP2, vimentin, and E-cadherin were evaluated in SH-eIF4E and control cells by IHC staining (200×, bar = 50 μm). (*P < 0.05, **P < 0.01, ***P < 0.001) (Student’s *t* test).

We further validated the effect of eIF4E on tumor progression and the relationship between eIF4E and markers of VM formation through in vivo animal experiments. As shown in Fig. S5, the immunohistochemical results of TA2 mouse tumor tissues were negative for ER-/PR-/HER2- expression. First, we used TA2 mice, which have spontaneous high-metastasis TNBC, and injected them with the SH-eIF4E plasmid. After injection with the SH-eIF4E plasmid, tumor growth in TA2 mice was slower than that in control TA2 mice (*P* < 0.01, Fig. 6A). In addition, the immunohistochemistry results indicated that the expression levels of vimentin and MMP2 were reduced in tumors with the SH-eIF4E plasmid and that the expression of E-cadherin was increased (*P* < 0.05, Fig. 6B). Together with the in vitro experiments, we conclude that eIF4E promotes TNBC development and the expression of EMT markers and MMP2 in vivo.

### The effect of RIPK1 on p-AKT/eIF4E, and VM formation-related and necroptosis-related markers in the TA2 mouse model

**Fig. 7:**
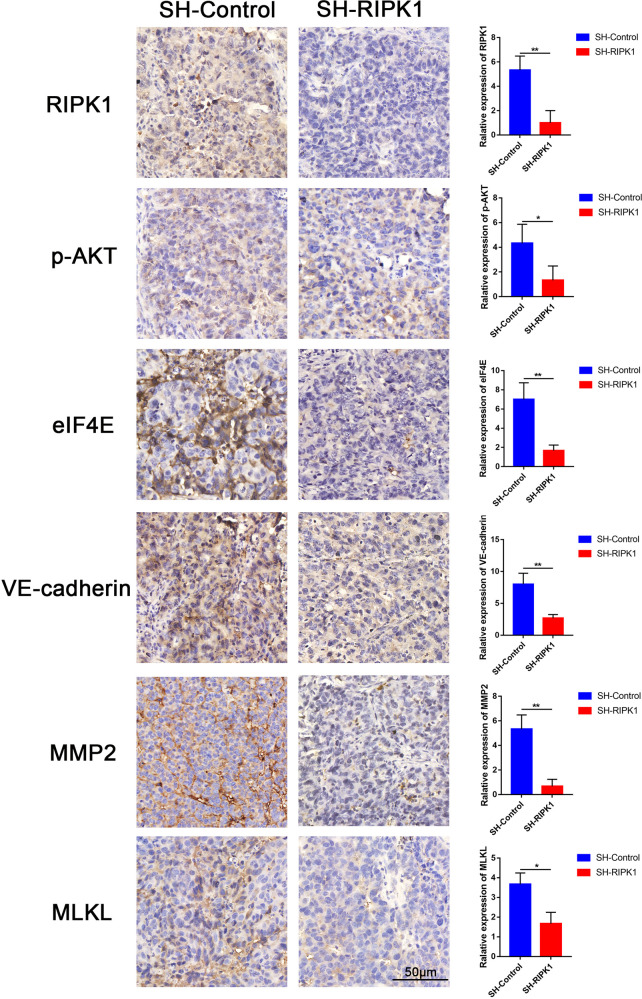
The effect of RIPK1 on p-AKT/eIF4E, and VM formation-related and necroptosis-related markers in the TA2 mouse model. The expression levels of RIPK1, p-AKT, eIF4E, VE-cadherin, MMP2, and MLKL were evaluated in SH-RIPK1 and control cells by IHC staining (200×, bar = 50 μm) (*P < 0.05, **P < 0.01) (Student’s *t* test).

We further performed immunohistochemistry using an in vivo-transfected RIPK1 knockdown vector in TA2 mice. Our results showed that tumor growth in TA2 mice injected with the SH-RIPK1 plasmid was slower than that in control TA2 mice (*P* = 0.016, Fig. S7). Our results showed that RIPK1 expression was significantly inhibited after transfection of the RIPK1-knockdown vector in TA2 mice compared with the negative control(*P* < 0.01, Fig. 7). In addition, the immunohistochemistry results indicated that the expression levels of p-AKT and eIF4E were reduced in tumors treated with the SH-RIPK1 plasmid compared with TA2 tumors in the negative control group, which was consistent with observations made in vitro. Immunohistochemical analysis of VE-cadherin and MMP2, which are established markers of VM formation, showed that VE-cadherin and MMP2 staining were the strongest in control mice and was significantly weaker in RIPK1-knockdown mice (*P* < 0.01, Fig. 7). MLKL is an established marker of necroptosis. MLKL staining was the strongest in control mice and was significantly weaker in RIPK1-knockdown mice (*P* < 0.05, Fig. 7), suggesting a reduction in necroptosis after knockdown of RIPK1 in TNBC. Thus, RIPK1 can promote p-AKT/eIF4E, and VM formation-related and necroptosis-related markers in the TA2 mouse model.

### Bioinformatics analysis of the clinical significance of eIF4E expression in BC

Analysis of The Cancer Genome Atlas (TCGA) RNA expression data and clinical case parameters showed that in the group of BC patients with high eIF4E expression, lymph node metastasis occurred in 71.3% of cases, including 244 cases in N1 (22.8%), 81 cases in N2 (7.6%) and 41 cases in N3 (3.8%), while only 17.6% of the low expression group had metastasis, which was statistically significant (*P* = 0.021) (Table 1).

**Table 1:** Correlation between eIF4E expression and clinicopathological parameters in TCGA breast cancer cases.

Characteristic	Low expression of eIF4E (%)	High expression of eIF4E (%)	*P* value
Age, *n* (%)			0.754
≤60	226 (20.5)	384 (34.8)	–
>60	178 (16.1)	315 (28.6)	–
T stage, *n* (%)			0.670
T1	100 (9)	181 (16.5)	–
T2	232 (21.1)	409 (37.2)	–
T3	57 (5.2)	81 (7.4)	–
T4	14 (1.3)	26 (2.4)	–
N stage, *n* (%)			0.021*
N0	208 (19.5)	307 (28.7)	–
N1	118 (11)	244 (22.8)	–
N2	36 (3.4)	81 (7.6)	–
N3	34 (3.2)	41 (3.8)	–
M stage, *n* (%)			0.367
M0	318 (34.1)	593 (63.6)	–
M1	10 (1.1)	12 (1.3)	–
Pathologic stage, *n* (%)			0.110
Stage I	75 (7)	104 (9.7)	–
Stage II	223 (20.9)	401 (37.5)	–
Stage III	88 (8.2)	161 (15.1)	–
Stage IV	10 (0.9)	7 (0.7)	–

^*^P < 0.05.

To further validate the correlation of eIF4E expression with VM formation-related molecules, we used the TCGA BC data and the GEO TNBC dataset GSE157284. Fig. S6A shows that in BC, eIF4E expression was significantly and positively correlated with expression of RIPK1 (*R* = 0.385, *P* < 0.001), RIPK3 expression (*R* = 0.068, *P* = 0.024) and AKT (*R* = 0.126, *P* < 0.001). In TNBC, eIF4E expression was significantly positively correlated with AKT (*R* = 0.48, *P* < 0.05), the EMT-related molecule vimentin (VIM) (*R* = 0.46, *P* < 0.05), the VM formation-related molecule MMP2 (*R* = 0.51, *P* < 0.05), and the necroptosis-related molecule MLKL (*R* = 0.306, *P* < 0.05) (Fig. S6B). Thus, necroptosis-related molecules were positively correlated with eIF4E expression, which was consistent with the other experimental results. The results indicate that eIF4E promotes VM formation.

## Discussion

Recent studies have revealed an important role of necroptosis in tumorigenesis and metastasis and indicate the potential of targeting necroptosis as a novel cancer therapy (necroptosis and tumor progression). In addition, necroptosis in cancer cells is considered to be an immunogenic cell death that can activate antitumor immunity. Yatim N et al. found that the induction of cancer cell necroptosis triggered the release of DAMPs to induce dendritic cell (DC) maturation [25]. Yang H et al. found that the deletion of RIPK3 or MLKL in cancer cells inhibited chemotherapy-induced immunogenic cell death (ICD) and anticancer immune responses in vivo [26]. Han-Hee Park et al. demonstrated that the RIPK1/RIPK3 signaling axis initiated TRIM28 derepression, which promoted DC maturation; TRIM28 overexpression impaired necroptosis-mediated cytokine production [27]. These results suggest that necroptosis can contribute to ICD signaling and tumor immunogenicity [28]. However, Zhaoshan Liu et al. found that necroptosis suppressed the antitumor activity of T cells in breast cancer, and the sE-cad/KLRG1 pathway played a major role in mediating necroptosis-mediated inhibition of the antitumor activity of T cells [29]. Additionally, necroptosis may promote the survival of circulating tumor cells (CTCs) by inhibiting the activity of peripheral blood T cells in an MVT-1 breast cancer model [29].

“Linear programmed necrosis” in non-small cell lung cancer induces tumor cells to die linearly and provides space for VM formation; second, it promotes the expression of MMP2, Twist1 and Slug, promoting extracellular matrix remodeling and EMT by DKK1, which in turn promotes VM formation [30]. Here, we investigated the effect of RIPK1-dependent necroptosis on VM formation in TNBC.

RIPK1 activates necroptosis signaling and contains an N-terminal kinase structural domain, a C-terminal death domain (DD) and an intermediate structural domain containing a RIP homotypic interaction motif (RHIM) [31]. The serine/threonine kinase activity of RIPK1 is essential for necroptosis [32]. Our results show that necroptosis is RIPK1-dependent in TNBC. It was found that the induction of necroptosis in endothelial cells promoted the extravasation and metastasis of tumor cells, and these effects were reduced by the RIPK1 inhibitor Nec-1 or specific knockdown of RIPK3 in endothelial cells [33]. We collected necroptotic conditioned medium and cocultured it with endothelial cells and BC cells to investigate its effect on tube formation. The results showed that necroptosis promoted endothelial cell tube and VM formation, which was inhibited by treatment with Nec-1 or knockdown of RIPK1. VM connectivity with endothelial vessels is essential for tumor cells to obtain a blood supply [6, 34], and our results demonstrate that necroptosis in tumor cells can promote tube formation by tumor cells and vascular endothelial cells, which in turn creates conditions for their connectivity and facilitates VM formation. VM provides oxygen and nutrients to rapidly growing tumors and serves as an escape route for metastasis [35, 36]. VE-cadherin is a major marker of VM and a key molecule in vascular endothelial cell-mediated adhesion junctions [10, 37]. Our results showed elevated VE-cadherin expression in TNBC cells during necroptosis, which demonstrated RIPK1-dependent features.

eIF4E is selectively involved in mRNA cap-dependent translation and nucleoplasmic transport [38, 39]. It has been demonstrated that elevated eIF4E levels in BC promote tumor progression [16]. Our analysis of TCGA clinicopathological data showed that high eIF4E expression in BC was significantly associated with tumor lymph node metastasis. Studies have shown that the kinase activity of RIPK1/3 promotes the transcription of inflammatory cytokines and that RIP1 drives the cap-dependent translation initiation pathway through the activation of eIF4E, which is the rate-limiting molecule of this pathway [40]. During RIPK1-dependent necroptosis, activation of eIF4E leads to increased expression of inflammatory factors [41]. We found elevated levels of eIF4E during necroptosis in TNBC cells. eIF4E levels were reduced after treatment with Nec-1 or knockdown of RIPK1. Regulation of eIF4E by the AKT pathway has been demonstrated in gastric, lung and ovarian cancers [42, 43]. Western blotting further verified that RIPK1 kinase could activate the AKT pathway. Our results showed that during necroptosis in TNBC cells, the p-AKT expression level was significantly increased. Chemical inhibition or knockdown of RIPK1 suppressed p-AKT expression. This finding suggests that phosphorylation and activation of AKT is RIPK1 dependent. During necroptosis, p-AKT and eIF4E levels were elevated, whereas eIF4E was suppressed after chemical inhibition of AKT. Studies have consistently shown that the phosphorylation and activation of AKT/mTOR in TNFα/zVAD-induced necroptosis in mouse neuronal cells is RIPK1-dependent [44]. Similarly, our results show that RIPK1 can promote p-AKT/eIF4E, and VM formation-related and necroptosis-related markers in the TA2 mouse model. Thus, during necroptosis in TNBC, RIPK1 promotes p-AKT/eIF4E signaling. Moreover, activation of the AKT-eIF4E pathway is dependent on RIPK1 and does not result from cell death. Increased expression of AKT-eIF4E is a consequence of RIPK1 regulation during necroptosis.

We further investigated the effect of eIF4E on VM formation. The results showed that high expression of eIF4E in BC cells promoted VM formation. Elvin Wagenblast et al. showed that the ability of breast tumor cells to metastasize was closely related to their ability to induce VM [45]. Tumor cell deformation and extracellular matrix remodeling are key components of VM formation, and EMT can help tumor cells regain plasticity and transform into mesenchymal morphology [46, 47]. In vivo animal experiments showed that knockdown of eIF4E expression significantly inhibited breast tumor growth volume, while immunohistochemistry showed that knockdown of eIF4E decreased the expression of the mesenchymal marker vimentin. In vivo and in vitro experiments showed that eIF4E could promote EMT, which in turn facilitated VM formation. Recently, evidence has indicated that the dynamic reciprocal transition between EMT and MET phenotypes represents the fundamental basis of tumor progression in TNBC [48]. The EMT and MET programs enable these cancer cells to dynamically change their phenotype, which is a feature that might be critical for successful metastatic dissemination and overcoming the multiple obstacles on route to a final destination-distant site parenchyma. EMT is considered an early event in breast cancer tumorigenesis, crucial for intravasation [48, 49]. MET, on the other hand, appears to be essential for subsequent reactivation in distant site and from the micro- to macro-metastatic stage [48, 50]. Multiple studies have also shown the involvement of hybrid E/M states in the aggressive behavior of TNBC [51, 52]. Three-dimensional culture of tumor cells and HUVECs showed that necroptotic conditioned medium promoted tumor cell and HUVEC tube formation, while knockdown of eIF4E blocked VM formation during necroptosis. Overexpression of eIF4E promoted tumor cell and HUVEC tube formation. Necroptosis in TNBC cells alters the composition of the extracellular environment, which in turn promotes VM. eIF4E expression is regulated by RIPK1 and promotes necroptosis-mediated VM formation. Thus, eIF4E is a key molecule in RIPK1-dependent necroptosis-mediated VM formation and could be a potential target for VM therapy.

Remodeling of tumor cells and the extracellular matrix to provide space for tumor cell deformation and motility is a major factor in VM formation, and MMP2 plays a key role in this process [53, 54]. MMP2 is a key mediator of invasion, metastasis, tumor angiogenesis and VM in tumor cells [55, 56]. Our results showed that in TNBC cells, MMP2 expression and enzyme activity were elevated during necroptosis. eIF4E expression leads to increased MMP2 expression. Knockdown of eIF4E blocked MMP2 expression during necroptosis. During RIPK1-dependent necroptosis, eIF4E promoted MMP2 expression and enzymatic activity, facilitating extracellular matrix degradation and providing the conditions for VM formation in TNBC. The in vitro immunohistochemistry results similarly showed that MMP2 expression decreased after the inhibition of eIF4E. Our results confirm that the activation of eIF4E signaling may be another important mechanism by which MMP2 mediates necroptosis-mediated VM. Our study provides a theoretical basis for necroptosis-mediated VM in TNBC.

## Conclusions

**Fig. 8:**
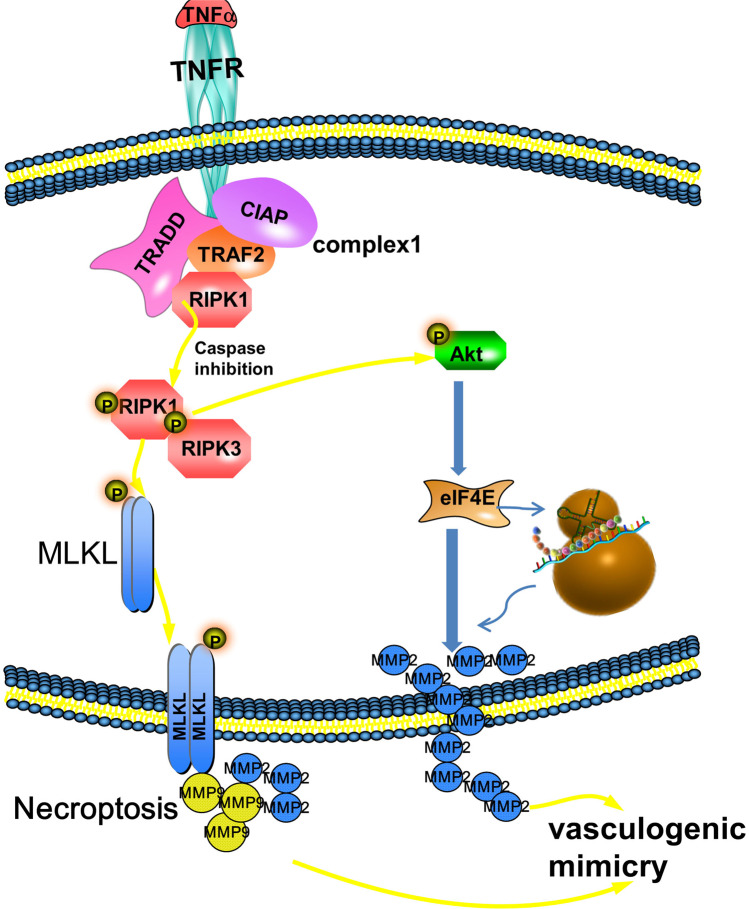
Schematic of the proposed mechanism by which the RIPK1-p-AKT-eIF4E-MMP2 interaction promotes VM during necroptosis inTNBC. During necroptosis in TNBC, the interaction of RIPK1-p-AKT promotes the expression of eIF4E, which promotes the expression and activity of MMP2, and MMP2 then induces VM formation.

RIPK1-dependent necroptosis promotes VM formation in TNBC. Necroptosis in TNBC promotes VM formation by activating RIPK1/p-AKT/eIF4E signaling, and eIF4E promotes EMT and MMP2 expression and activity, leading to VM formation (Fig. 8). Our study provides a rationale for necroptosis-mediated VM and also providing a potential therapeutic target for TNBC.

## Supplementary information

supplementary figure legends

Figure Supplemental 1

Figure Supplemental 2

Figure Supplemental 3

Figure Supplemental 4

Figure Supplemental 5

Figure Supplemental 6

Figure Supplemental 7

aj-checklist

Original Data File

## Data Availability

All data generated during this study are included in this published article and its supplementary information files. The TCGA breast cancer RNA expression data and clinical case parameters are available in the TCGA database (https://portal.gdc.cancer.gov). The GSE157284 datasets generated during and analyses during the current study are available in the GEO database(http://www.ncbi.nlm.nih.gov/geo/). The analysis during the study can be obtained from the corresponding author Danfang Zhang on reasonable request.

## References

[1] Lei S, Zheng R, Zhang S, Chen R, Wang S, Sun K (2021). Breast cancer incidence and mortality in women in China: temporal trends and projections to 2030. Cancer Biol Med.

[2] Sung H, Ferlay J, Siegel RL, Laversanne M, Soerjomataram I, Jemal A (2021). Global Cancer Statistics 2020: GLOBOCAN estimates of incidence and mortality worldwide for 36 cancers in 185 countries. CA: A Cancer J Clin.

[3] Bergin ART, Loi S (2019). Triple-negative breast cancer: recent treatment advances. F1000Research.

[4] Cao L, Niu Y (2020). Triple negative breast cancer: special histological types and emerging therapeutic methods. Cancer Biol Med.

[5] Majidpoor J, Mortezaee K (2021). Angiogenesis as a hallmark of solid tumors - clinical perspectives. Cell Oncol.

[6] Zhang S, Zhang D, Sun B (2007). Vasculogenic mimicry: current status and future prospects. Cancer Lett.

[7] Babaei G, Aziz SG, Jaghi NZZ (2021). EMT, cancer stem cells and autophagy; The three main axes of metastasis. Biomed Pharmacother Biomed Pharmacother.

[8] Zhao B, Wu M, Hu Z, Ma Y, Qi W, Zhang Y (2020). Thrombin is a therapeutic target for non-small-cell lung cancer to inhibit vasculogenic mimicry formation. Signal Transduct Target Ther.

[9] De Palma M, Biziato D, Petrova TV (2017). Microenvironmental regulation of tumour angiogenesis. Nat Rev Cancer.

[10] Yang J, Lu Y, Lin YY, Zheng ZY, Fang JH, He S (2016). Vascular mimicry formation is promoted by paracrine TGF-beta and SDF1 of cancer-associated fibroblasts and inhibited by miR-101 in hepatocellular carcinoma. Cancer Lett.

[11] Asleh K, Riaz N, Nielsen TO (2022). Heterogeneity of triple negative breast cancer: Current advances in subtyping and treatment implications. J Exp Clin Cancer Res.

[12] Xu D, Zou C, Yuan J (2021). Genetic regulation of RIPK1 and necroptosis. Annu Rev Genet.

[13] Amin P, Florez M, Najafov A, Pan H, Geng J, Ofengeim D (2018). Regulation of a distinct activated RIPK1 intermediate bridging complex I and complex II in TNFalpha-mediated apoptosis. Proc Natl Acad Sci USA.

[14] Zhou W, Yuan J (2014). Necroptosis in health and diseases. Semin Cell Dev Biol.

[15] Degterev A, Hitomi J, Germscheid M, Ch'en IL, Korkina O, Teng X (2008). Identification of RIP1 kinase as a specific cellular target of necrostatins. Nat Chem Biol.

[16] Li F, Sun H, Li Y, Bai X, Dong X, Zhao N (2021). High expression of eIF4E is associated with tumor macrophage infiltration and leads to poor prognosis in breast cancer. BMC Cancer.

[17] Carroll M, Borden KL (2013). The oncogene eIF4E: using biochemical insights to target cancer. J Interferon Cytokine Res.

[18] Siddiqui N, Sonenberg N (2015). Signalling to eIF4E in cancer. Biochem Soc Trans.

[19] Bhat M, Robichaud N, Hulea L, Sonenberg N, Pelletier J, Topisirovic I (2015). Targeting the translation machinery in cancer. Nat Rev Drug Discov.

[20] Seifert L, Werba G, Tiwari S, Giao Ly NN, Alothman S, Alqunaibit D (2016). The necrosome promotes pancreatic oncogenesis via CXCL1 and Mincle-induced immune suppression. Nature.

[21] Akara-Amornthum P, Lomphithak T, Choksi S, Tohtong R, Jitkaew S (2020). Key necroptotic proteins are required for Smac mimetic-mediated sensitization of cholangiocarcinoma cells to TNF-alpha and chemotherapeutic gemcitabine-induced necroptosis. PLoS ONE.

[22] Chen D, Yu J, Zhang L (2016). Necroptosis: an alternative cell death program defending against cancer. Biochim. Biophys. Acta.

[23] Sun L, Wang H, Wang Z, He S, Chen S, Liao D (2012). Mixed lineage kinase domain-like protein mediates necrosis signaling downstream of RIP3 kinase. Cell.

[24] Pawlikowska M, Jedrzejewski T, Brozyna AA, Wrotek S (2020). Protein-bound polysaccharides from coriolus versicolor induce RIPK1/RIPK3/MLKL-mediated necroptosis in ER-positive breast cancer and amelanotic melanoma cells. Cell Physiol Biochem: Int J Exp Cell Physiol Biochem Pharmacol.

[25] Yatim N, Jusforgues-Saklani H, Orozco S, Schulz O, Barreira da Silva R (2015). Reis e Sousa C, et al. RIPK1 and NF-kappaB signaling in dying cells determines cross-priming of CD8(+) T cells. Science.

[26] Yang H, Ma Y, Chen G, Zhou H, Yamazaki T, Klein C (2016). Contribution of RIP3 and MLKL to immunogenic cell death signaling in cancer chemotherapy. Oncoimmunology.

[27] Snyder AG, Hubbard NW, Messmer MN, Kofman SB, Hagan CE, Orozco SL (2019). Intratumoral activation of the necroptotic pathway components RIPK1 and RIPK3 potentiates antitumor immunity. Sci Immunol.

[28] Zhu F, Zhang W, Yang T, He SD (2019). Complex roles of necroptosis in cancer. J Zhejiang Univ Sci B.

[29] Liu Z, Choksi S, Kwon HJ, Jiao D, Liu C, Liu ZG (2023). Tumor necroptosis-mediated shedding of cell surface proteins promotes metastasis of breast cancer by suppressing anti-tumor immunity. Breast Cancer Res.

[30] Yao L, Zhang D, Zhao X, Sun B, Liu Y, Gu Q (2016). Dickkopf-1-promoted vasculogenic mimicry in non-small cell lung cancer is associated with EMT and development of a cancer stem-like cell phenotype. J Cell Mol Med.

[31] Holler N, Zaru R, Micheau O, Thome M, Attinger A, Valitutti S (2000). Fas triggers an alternative, caspase-8-independent cell death pathway using the kinase RIP as effector molecule. Nat Immunol.

[32] Christofferson DE, Yuan J (2010). Necroptosis as an alternative form of programmed cell death. Curr Opin Cell Biol.

[33] Strilic B, Yang L, Albarrán-Juárez J, Wachsmuth L, Han K, Müller UC (2016). Tumour-cell-induced endothelial cell necroptosis via death receptor 6 promotes metastasis. Nature.

[34] Sood AK, Fletcher MS, Coffin JE, Yang M, Seftor EA, Gruman LM (2004). Functional role of matrix metalloproteinases in ovarian tumor cell plasticity. Am J Obstet Gynecol.

[35] Shirakawa K, Kobayashi H, Heike Y, Kawamoto S, Brechbiel MW, Kasumi F (2002). Hemodynamics in vasculogenic mimicry and angiogenesis of inflammatory breast cancer xenograft. Cancer Res.

[36] Clarijs R, Otte-Holler I, Ruiter DJ, de Waal RM (2002). Presence of a fluid-conducting meshwork in xenografted cutaneous and primary human uveal melanoma. Investig Ophthalmol Vis Sci.

[37] Kirschmann DA, Seftor EA, Hardy KM, Seftor RE, Hendrix MJ (2012). Molecular pathways: vasculogenic mimicry in tumor cells: diagnostic and therapeutic implications. Clin Cancer Res.

[38] Wu M, Liu Y, Di X, Kang H, Zeng H, Zhao Y (2013). EIF4E over-expresses and enhances cell proliferation and cell cycle progression in nasopharyngeal carcinoma. Med Oncol.

[39] Volpon L, Osborne MJ, Borden KLB (2019). Biochemical and structural insights into the eukaryotic translation initiation factor eIF4E. Curr Protein Pept Sci.

[40] Najjar M, Saleh D, Zelic M, Nogusa S, Shah S, Tai A (2016). RIPK1 and RIPK3 kinases promote cell-death-independent inflammation by toll-like receptor 4. Immunity.

[41] Muendlein HI, Sarhan J, Liu BC, Connolly WM, Schworer SA, Smirnova I (2020). Constitutive interferon attenuates RIPK1/3-mediated cytokine translation. Cell Rep.

[42] Riquelme I, Tapia O, Espinoza JA, Leal P, Buchegger K, Sandoval A (2016). The gene expression status of the PI3K/AKT/mTOR pathway in gastric cancer tissues and cell lines. Pathol Oncol Res.

[43] Yoshizawa A, Fukuoka J, Shimizu S, Shilo K, Franks TJ, Hewitt SM (2010). Overexpression of phospho-eIF4E is associated with survival through AKT pathway in non-small cell lung cancer. Clin Cancer Res.

[44] Liu Q, Qiu J, Liang M, Golinski J, van Leyen K, Jung JE (2014). Akt and mTOR mediate programmed necrosis in neurons. Cell Death Dis.

[45] Wagenblast E, Soto M, Gutiérrez-Ángel S, Hartl CA, Gable AL, Maceli AR (2015). A model of breast cancer heterogeneity reveals vascular mimicry as a driver of metastasis. Nature.

[46] Gong W, Sun B, Zhao X, Zhang D, Sun J, Liu T (2016). Nodal signaling promotes vasculogenic mimicry formation in breast cancer via the Smad2/3 pathway. Oncotarget.

[47] Zhou J, Zhu X, Wu S, Guo J, Zhang K, Xu C (2020). Epithelial-mesenchymal transition status of circulating tumor cells in breast cancer and its clinical relevance. Cancer Biol Med.

[48] Kvokackova B, Remsik J, Jolly MK, Soucek K (2021). Phenotypic heterogeneity of triple-negative breast cancer mediated by epithelial-mesenchymal plasticity. Cancers.

[49] Ye X, Tam WL, Shibue T, Kaygusuz Y, Reinhardt F, Ng Eaton E (2015). Distinct EMT programs control normal mammary stem cells and tumour-initiating cells. Nature.

[50] Brabletz T (2012). To differentiate or not-routes towards metastasis. Nat Rev Cancer.

[51] Yoshida T, Ozawa Y, Kimura T, Sato Y, Kuznetsov G, Xu S (2014). Eribulin mesilate suppresses experimental metastasis of breast cancer cells by reversing phenotype from epithelial-mesenchymal transition (EMT) to mesenchymal-epithelial transition (MET) states. Br J Cancer.

[52] Yamamoto M, Sakane K, Tominaga K, Gotoh N, Niwa T, Kikuchi Y (2017). Intratumoral bidirectional transitions between epithelial and mesenchymal cells in triple-negative breast cancer. Cancer Sci.

[53] Sun B, Zhang S, Zhang D, Du J, Guo H, Zhao X (2006). Vasculogenic mimicry is associated with high tumor grade, invasion and metastasis, and short survival in patients with hepatocellular carcinoma. Oncol Rep.

[54] Liang X, Sun R, Zhao X, Zhang Y, Gu Q, Dong X (2017). Rictor regulates the vasculogenic mimicry of melanoma via the AKT-MMP-2/9 pathway. J Cell Mol Med.

[55] Chang C, Werb Z (2001). The many faces of metalloproteases: cell growth, invasion, angiogenesis and metastasis. Trends Cell Biol.

[56] Chen LX, He YJ, Zhao SZ, Wu JG, Wang JT, Zhu LM (2011). Inhibition of tumor growth and vasculogenic mimicry by curcumin through down-regulation of the EphA2/PI3K/MMP pathway in a murine choroidal melanoma model. Cancer Biol Ther.

