# Correction: RIPK1-dependent necroptosis promotes vasculogenic mimicry formation via eIF4E in triple-negative breast cancer

**DOI:** 10.1038/s41419-023-06052-z

**Published:** 2023-09-14

**Authors:** Fan Li, Huizhi Sun, Yihui Yu, Na Che, Jiyuan Han, Runfen Cheng, Nan Zhao, Yuhong Guo, Chongbiao Huang, Danfang Zhang

**Affiliations:** 1 Department of Pathology, Tianjin Medical University, Tianjin 300070 Tianjin, China; 2 Tianjin Medical University Cancer Institute and Hospital, National Clinical Research Center for Cancer, Key Laboratory of Cancer Prevention and Therapy, Tianjin’s Clinical Research Center for Cancer, 300060 Tianjin, China; 3 Department of Pathology, Tianjin Medical University General Hospital, 300052 Tianjin, China

**Keywords:** Tumour angiogenesis, Necroptosis

Correction to: *Cell Death and Disease* (2023) 14:335 10.1038/s41419-023-05841-w, published online 22 May 2023

In this article the affiliations have been given erroneously. They should be read:

1. Department of Pathology, Tianjin Medical University, Tianjin 300070, China

2. Tianjin Medical University Cancer Institute and Hospital, National Clinical Research Center for Cancer, Key Laboratory of Cancer Prevention and Therapy, Tianjin’s Clinical Research Center for Cancer, 300060, Tianjin, China

The original article has been corrected.

