# Supplementary material for: RIPK1-dependent necroptosis promotes vasculogenic mimicry formation via eIF4E in triple-negative breast cancer
Source: Cell Death Dis. 2023 May 22;14(5):335. doi: 10.1038/s41419-023-05841-w (PMC10203343; doi:10.1038/s41419-023-05841-w)
Supplement: Supplementary file 1 — supplementary figure legends [file 41419_2023_5841_MOESM1_ESM.docx]

**Figure S1.** Expression of RIPK1 in breast cancer cells and after transfection with SH-RIPK1 and EX-RIPK1 plasmids. A Western blotting and quantitative analysis (B) of MDA-MB-231 and MCF-7 cells. C, D The transfection efficiency of RIPK1. Knockdown of RIPK1 was mediated by infecting MDA-MB-231 cells with lentiviruses expressing specific shRNAs, the RIPK1 overexpression plasmid was transfected into MCF-7 cells, and Western blotting was used to evaluate the transfection efficiency. GAPDH was used as a loading control. Data are representative of three independent experiments (* *P* < 0.05, ** *P* < 0.01) (Student’S t test).

**Figure S2.** A Necroptosis cells were increased after treatment with TNF/Z-VAD (TZ) and TNF/Z-VAD/AZD5528 (TZA), but not TNFα alone or Z-VAD/AZD5528 (ZA) in Hs578T and MDA-MB-231 cells. Cells were treated with TNF (30 ng/ml)/z-VAD-fmk(20 µM)/AZD5528 (TZA), TNF/Z-VAD (TZ), Z-VAD/ AZD5528 (ZA) or TNFα alone and cell death was determined by fluorescence microscopy. red: PI (propidium iodide), blue: DAPI. PI^+^/DAPI staining was used to count the cell death value. (* *P* < 0.05, ** *P* < 0.01, *** *P* < 0.001, **** *P* < 0.0001, bar = 200 μm) B Necroptotic Hs578T cell-conditioned medium promoted three-dimensional tube formation in HUVECs and Hs578T cells, and RIPK1 inhibitors blocked TZA-induced tube formation. Data are representative of three independent experiments * *P* < 0.05, ** *P* < 0.01, *** *P* < 0.001, **** *P* < 0.0001 (one-way ANOVA).

**Figure S3. A.** Western blot analysis of VE-cadherin and p-AKT/eIF4E signals after T, TZ, ZA and TZA stimulations in MDA-MB-231 cells. B. Western blot analysis of p-AKT/eIF4E signals after GSK’872 (10 µM) for 2 h or NSA (1.5 µM) treatment for 12 h in response to TZA in MDA-MB-231 and Hs578T cells. Data are representative of three independent experiments. * *P* < 0.05, ** *P* < 0.01, *** *P* < 0.001 (one-way ANOVA).

**Figure S4.** Expression of eIF4E in breast cancer cells and after transfection with SH-eIF4E and EX-eIF4E plasmids. A Western blotting and quantitative analysis (B) of MDA-MB-231 and MCF-7 cells. C, D The transfection efficiency of eIF4E. Knockdown of eIF4E was mediated by infecting MDA-MB-231 cells with lentiviruses expressing the specific shRNAs, eIF4E overexpression plasmids were transfected into MCF-7 cells, and Western blotting was used to evaluate the transfection efficiency. GAPDH was used as a loading control. Data are representative of three independent experiments (* *P* < 0.05, ** *P* < 0.01) (Student’S t test).

**Figure S5.** TA2 mouse tumor tissues were negative for ER-/PR-/HER2- expression.

**Figure S6.** Analysis of the relationship between eIF4E and molecules associated with VM formation in the TCGA-BRCA database (A) and GEO-TNBC dataset (B).

**Figure S7.** Tumor growth curves showed that SH-RIPK1 inhibited tumor growth in the TA2 mouse model. (* *P* < 0.05) (Student’S t test)
