# Supplementary figures and images for: RIPK1-dependent necroptosis promotes vasculogenic mimicry formation via eIF4E in triple-negative breast cancer
Source: Cell Death Dis. 2023 May 22;14(5):335. doi: 10.1038/s41419-023-05841-w (PMC10203343; doi:10.1038/s41419-023-05841-w)

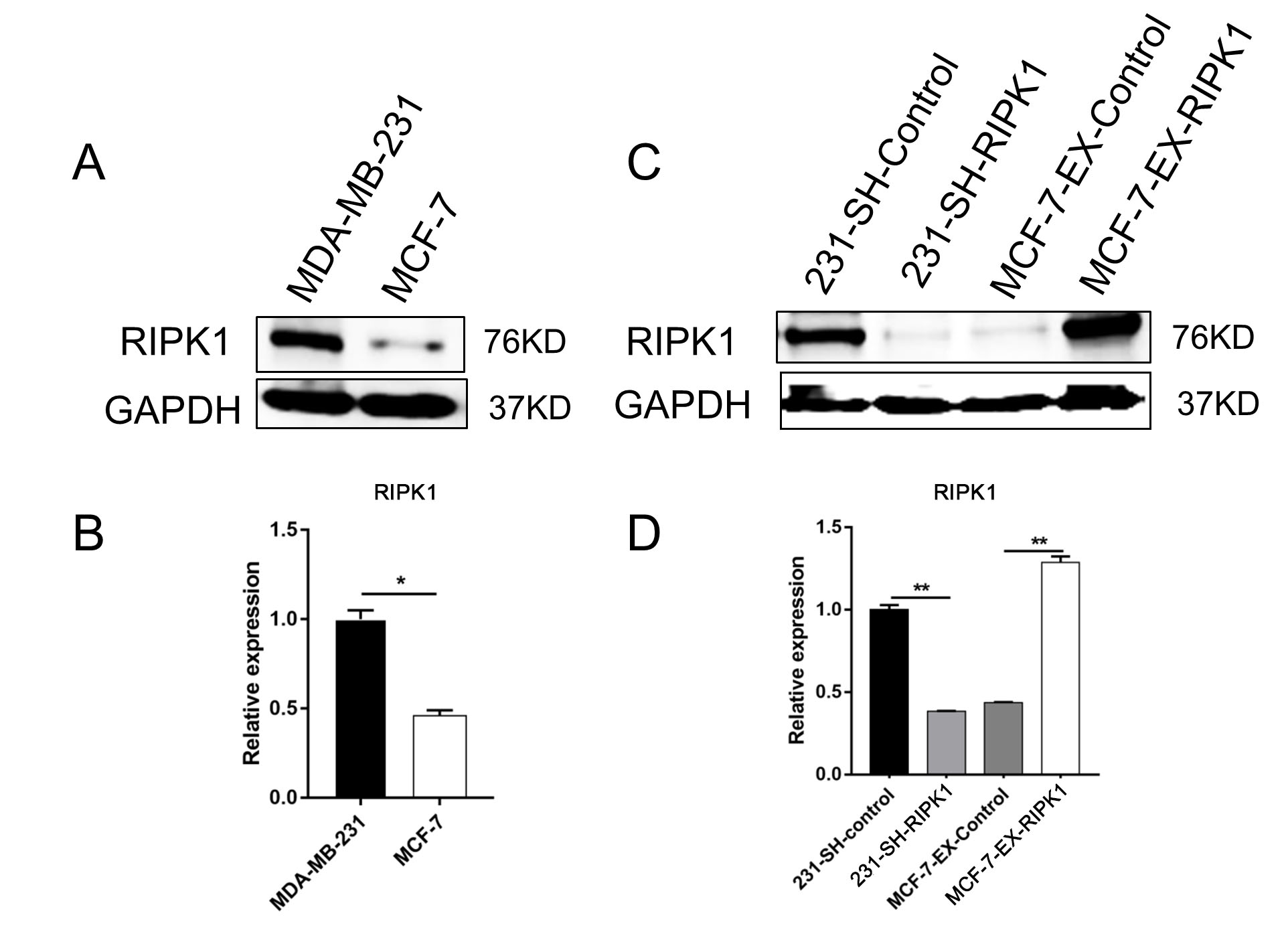

Supplement: Supplementary file 2 — Figure Supplemental 1 [file 41419_2023_5841_MOESM2_ESM.jpg]

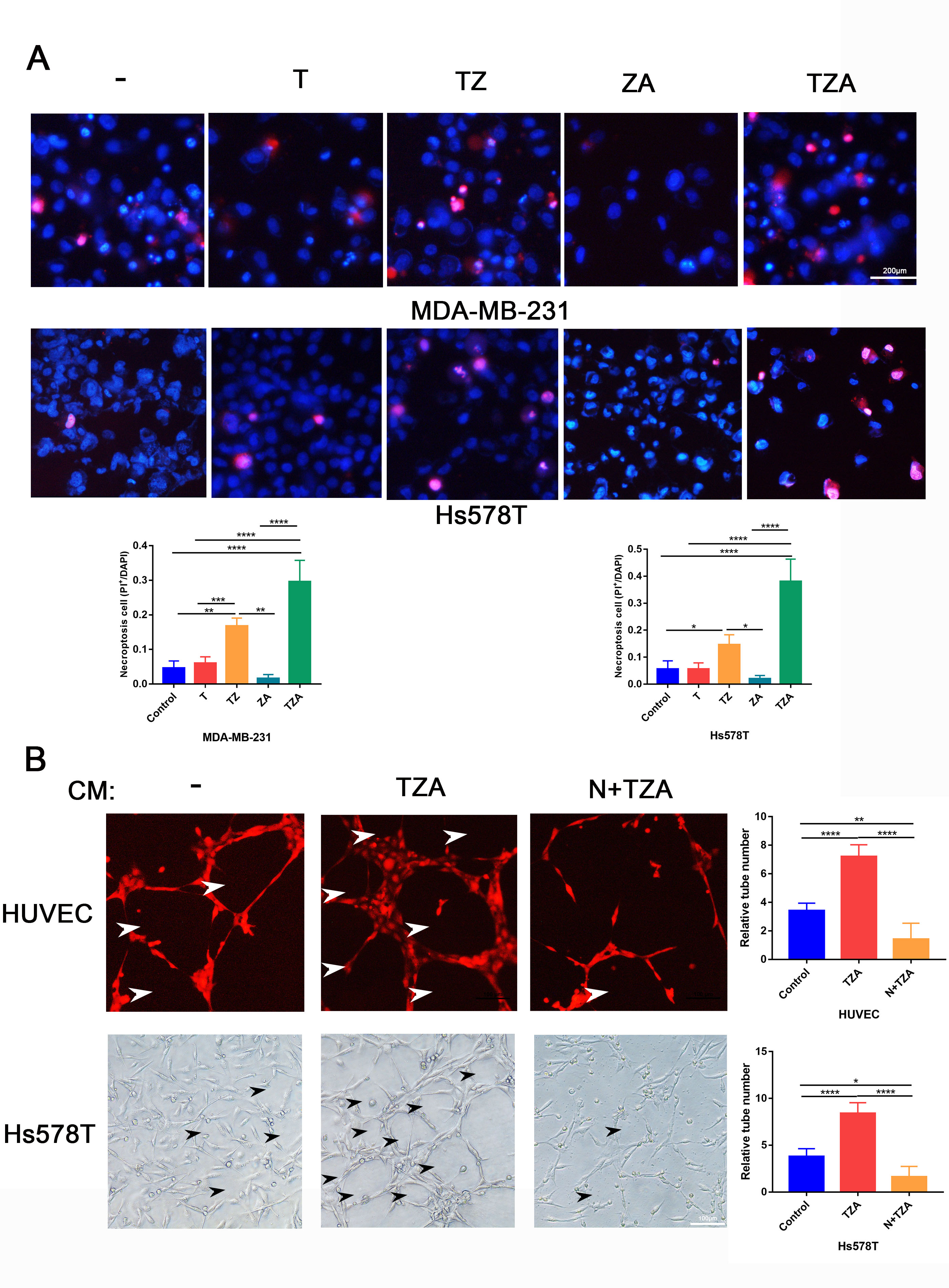

Supplement: Supplementary file 3 — Figure Supplemental 2 [file 41419_2023_5841_MOESM3_ESM.jpg]

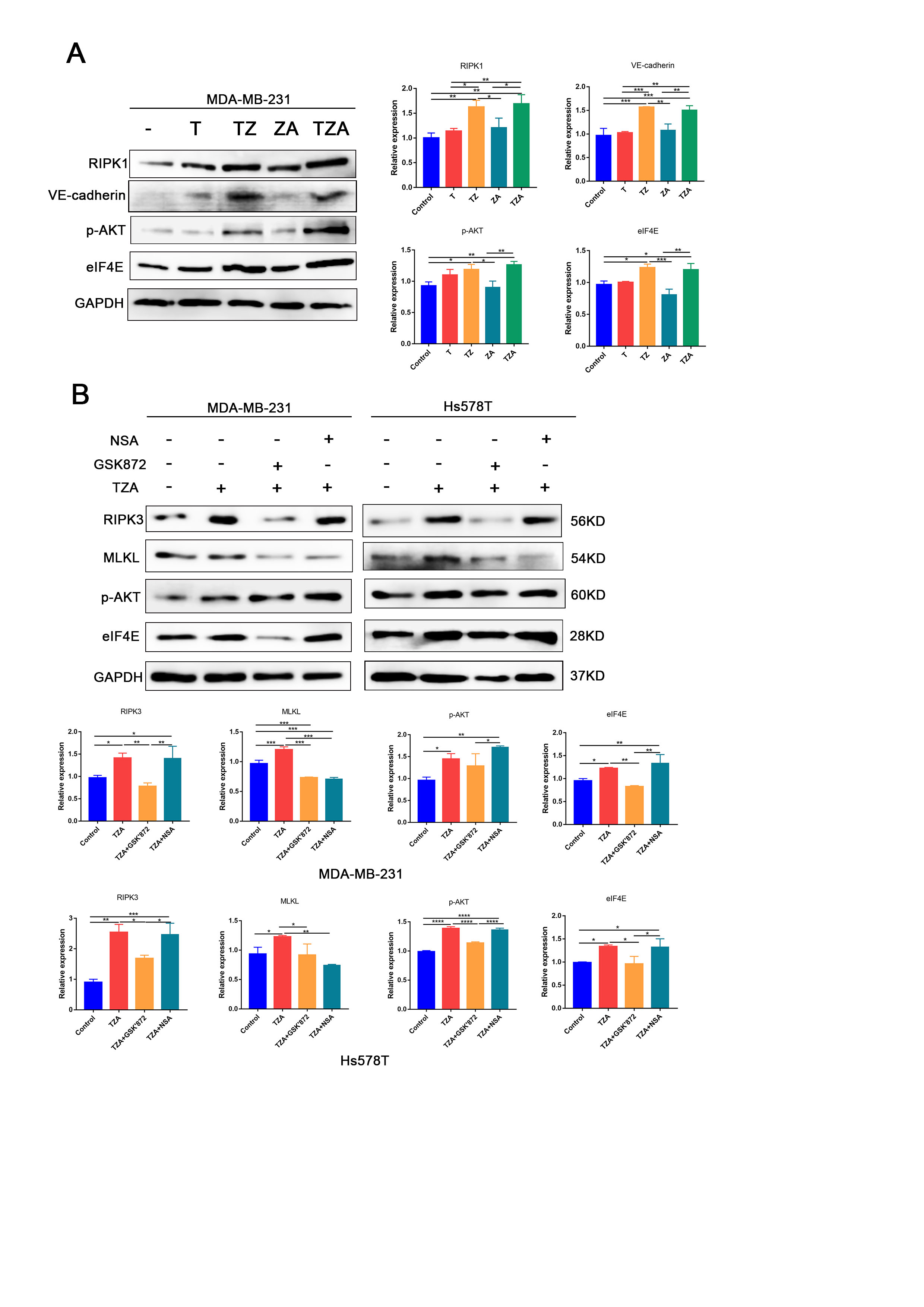

Supplement: Supplementary file 4 — Figure Supplemental 3 [file 41419_2023_5841_MOESM4_ESM.jpg]

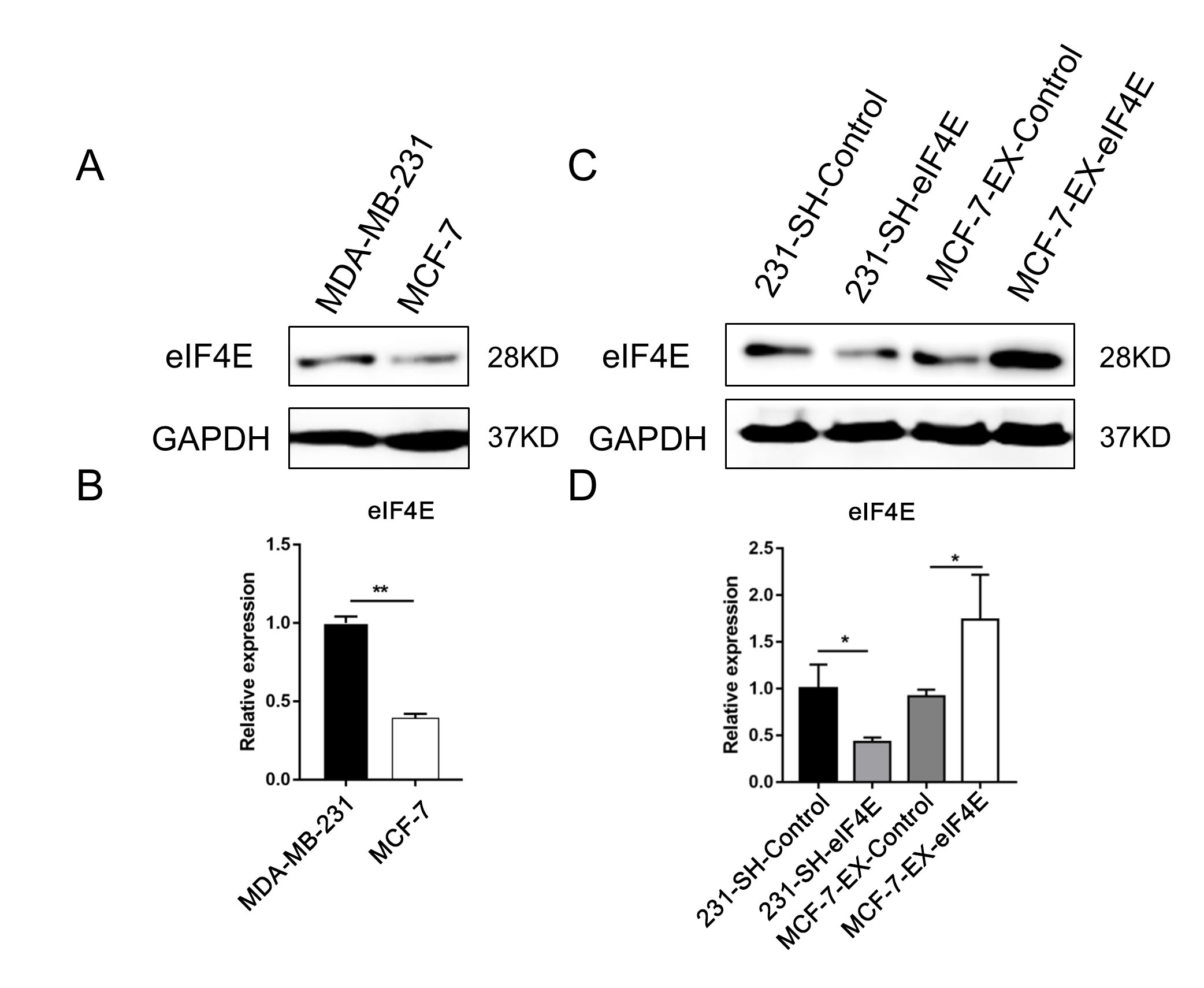

Supplement: Supplementary file 5 — Figure Supplemental 4 [file 41419_2023_5841_MOESM5_ESM.jpg]

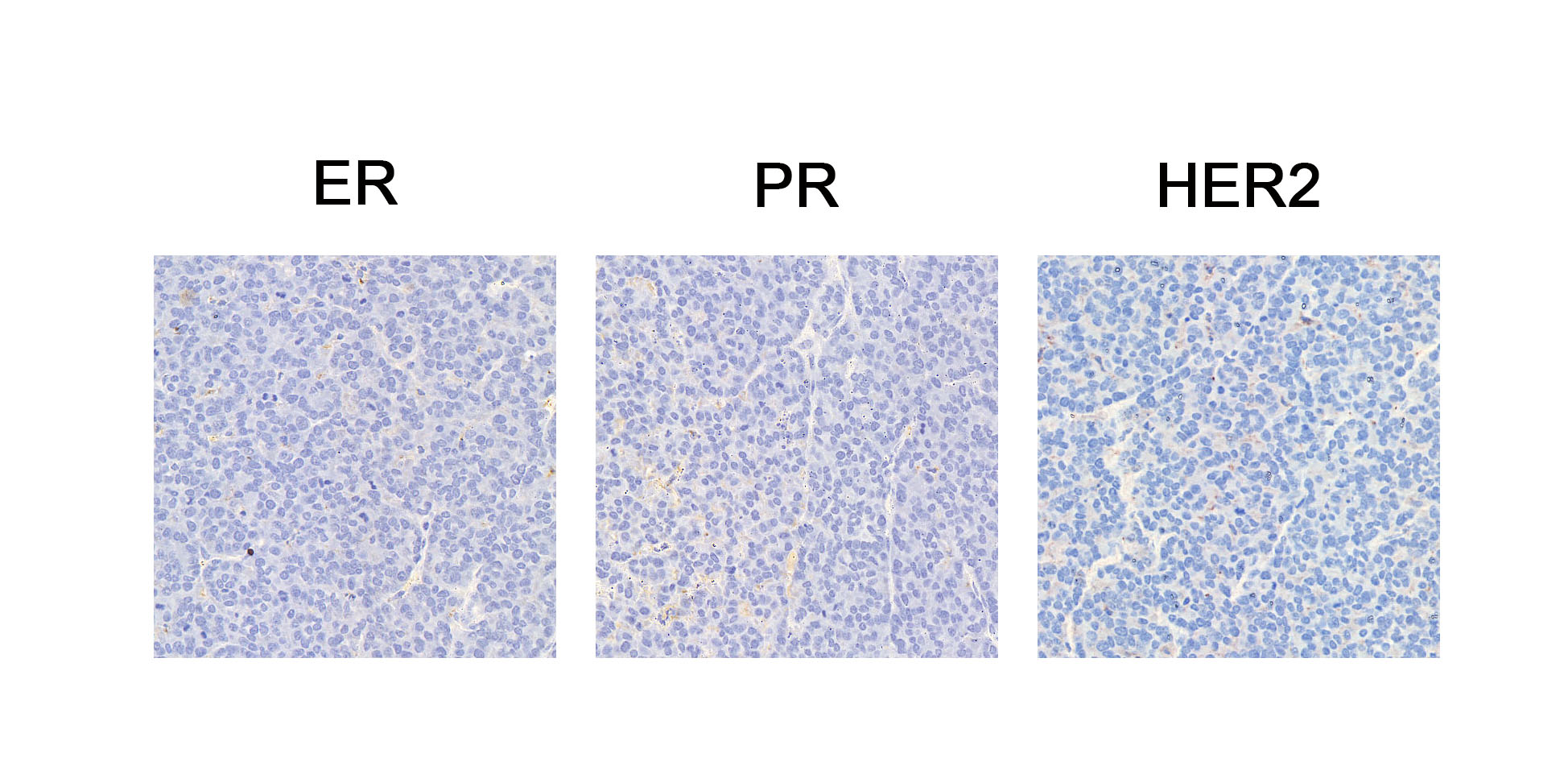

Supplement: Supplementary file 6 — Figure Supplemental 5 [file 41419_2023_5841_MOESM6_ESM.jpg]

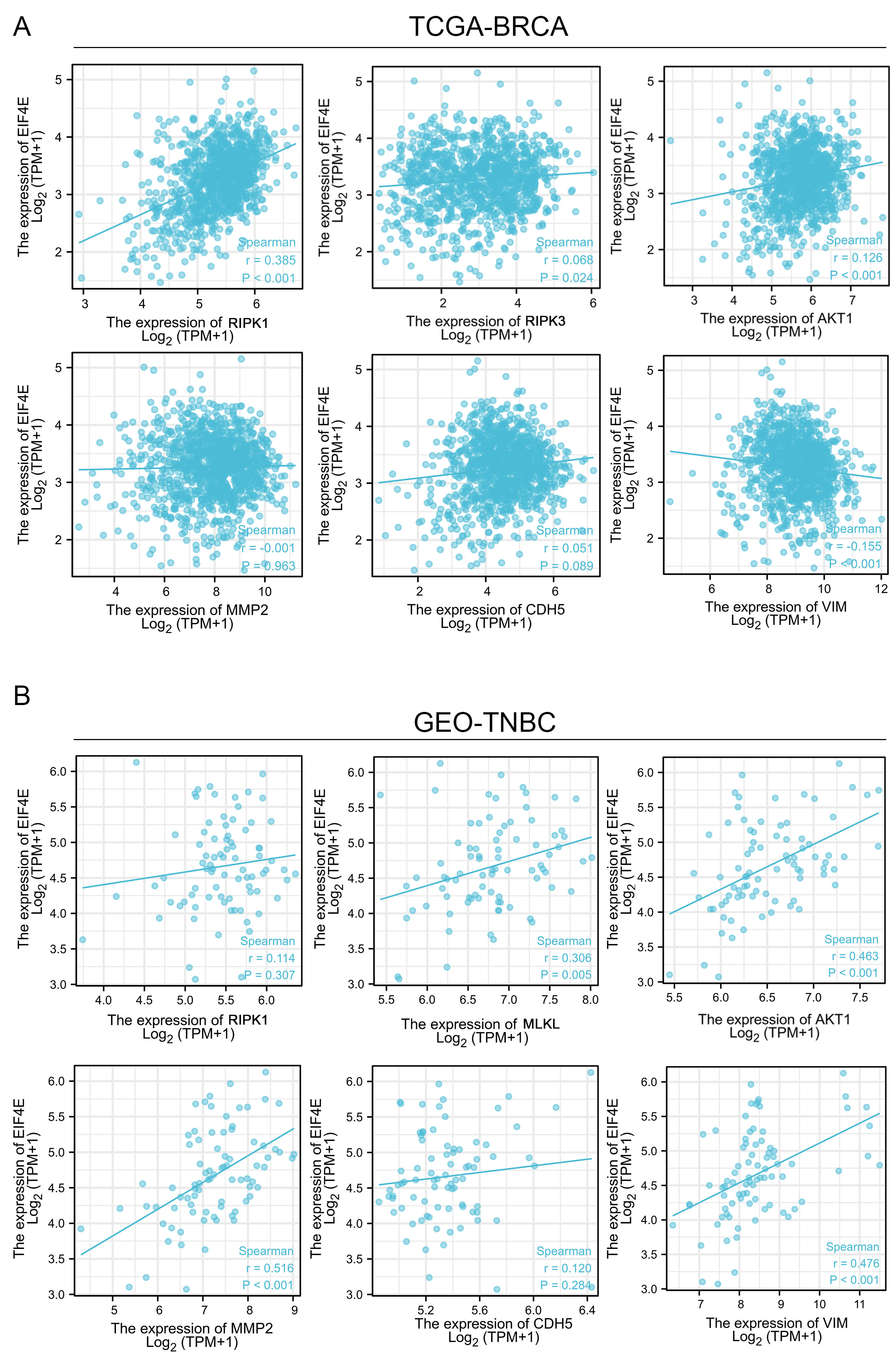

Supplement: Supplementary file 7 — Figure Supplemental 6 [file 41419_2023_5841_MOESM7_ESM.jpg]

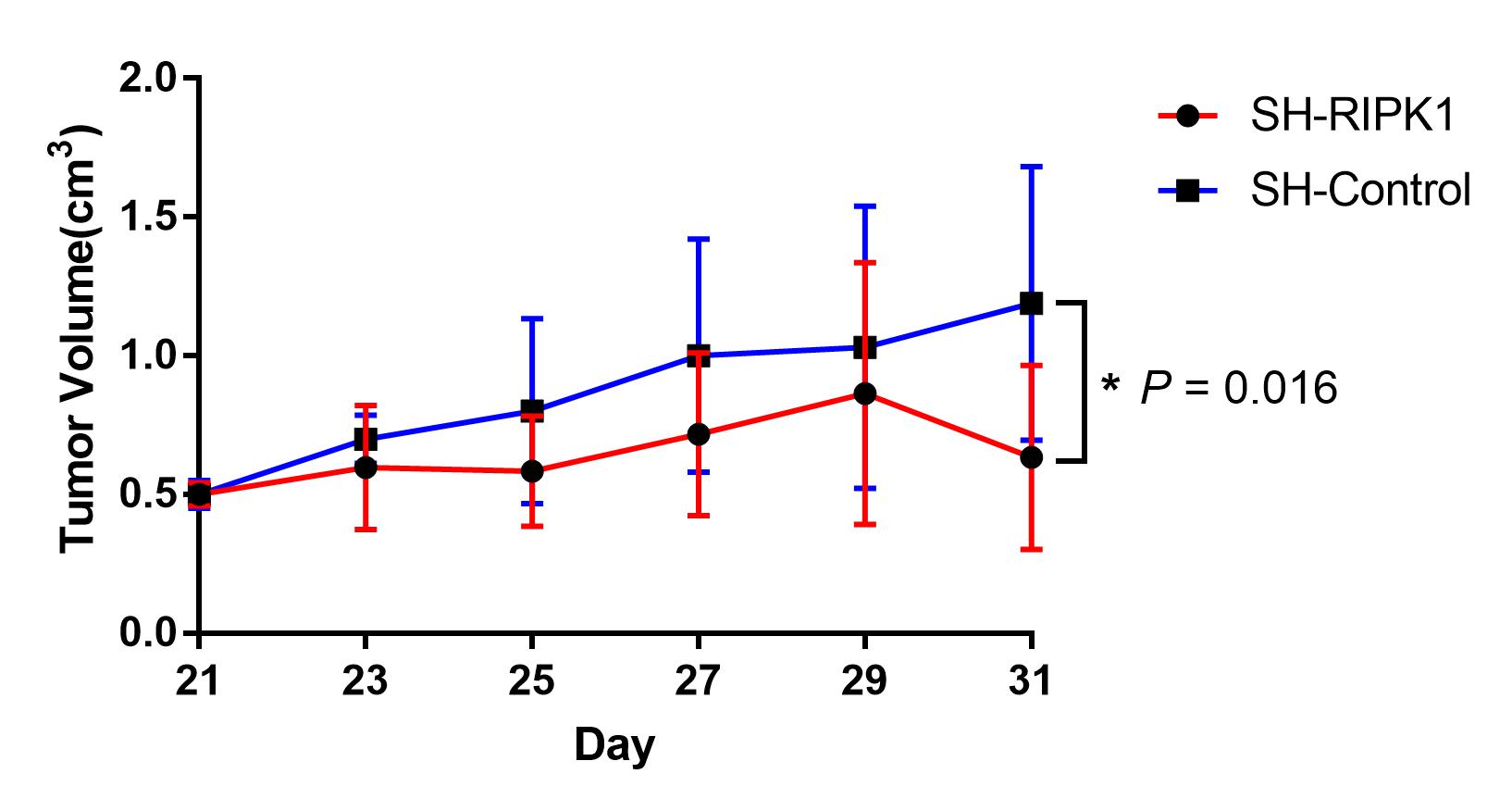

Supplement: Supplementary file 8 — Figure Supplemental 7 [file 41419_2023_5841_MOESM8_ESM.jpg]
