# Supplementary material for: RIPK1-dependent necroptosis promotes vasculogenic mimicry formation via eIF4E in triple-negative breast cancer
Source: Cell Death Dis. 2023 May 22;14(5):335. doi: 10.1038/s41419-023-05841-w (PMC10203343; doi:10.1038/s41419-023-05841-w)

Figure 1G

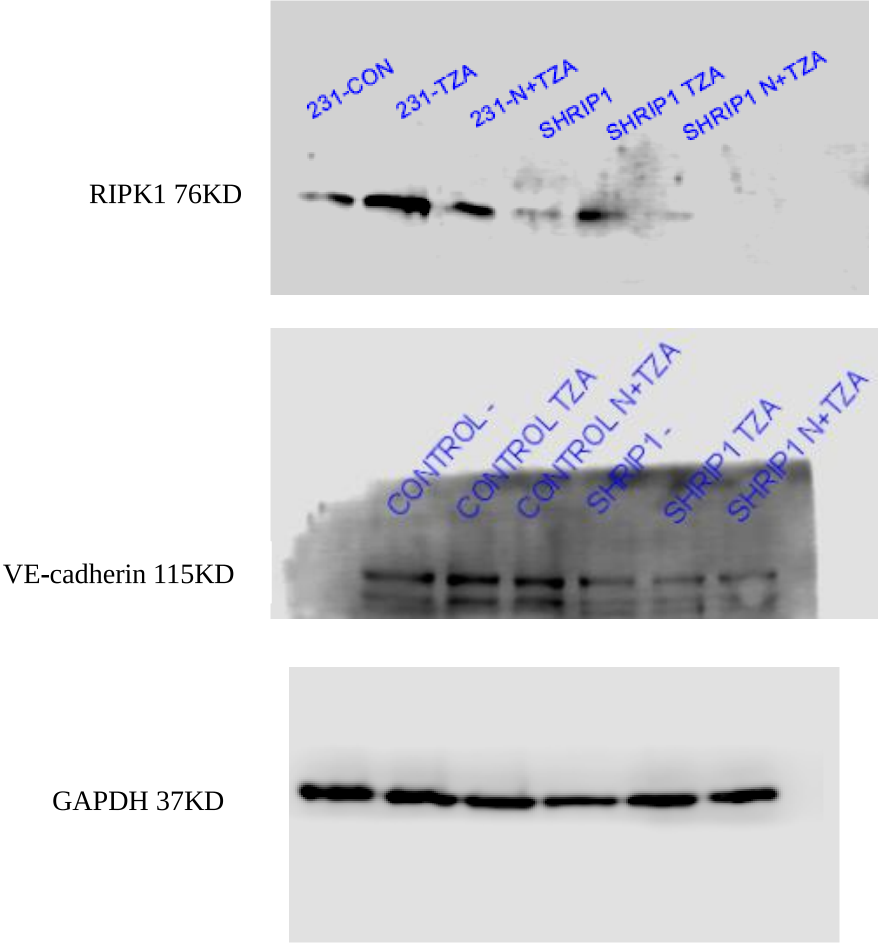
231-CON
231-TZA
231-N+TZA
SHRIP1
SHRIP1 TZA
SHRIP1 N+TZA
RIPK1 76KD
CONTROL -
CONTROL TZA
CONTROL N+TZA
SHRIP1 -
SHRIP1 TZA
SHRIP1 N+TZA
VE-cadherin 115KD
GAPDH 37KD

Figure 1I

RIPK1 76KD

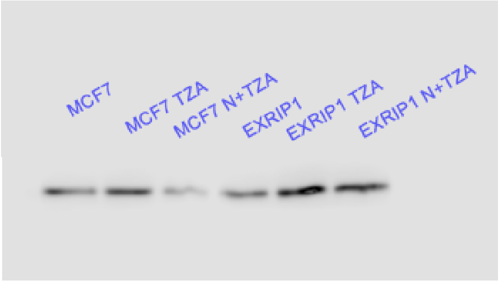

VE-cadherin 115KD

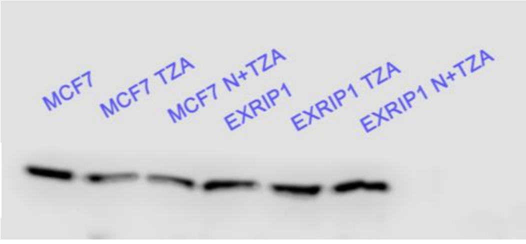

GAPDH 37KD

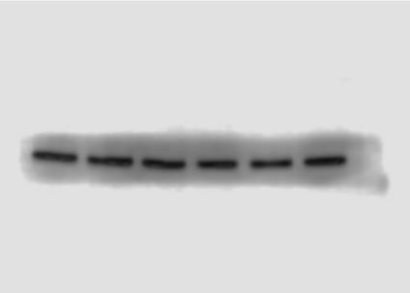

Figure 2A

eIF4E 28KD

AKT 55KD

p-AKT 60KD

GAPDH 37KD

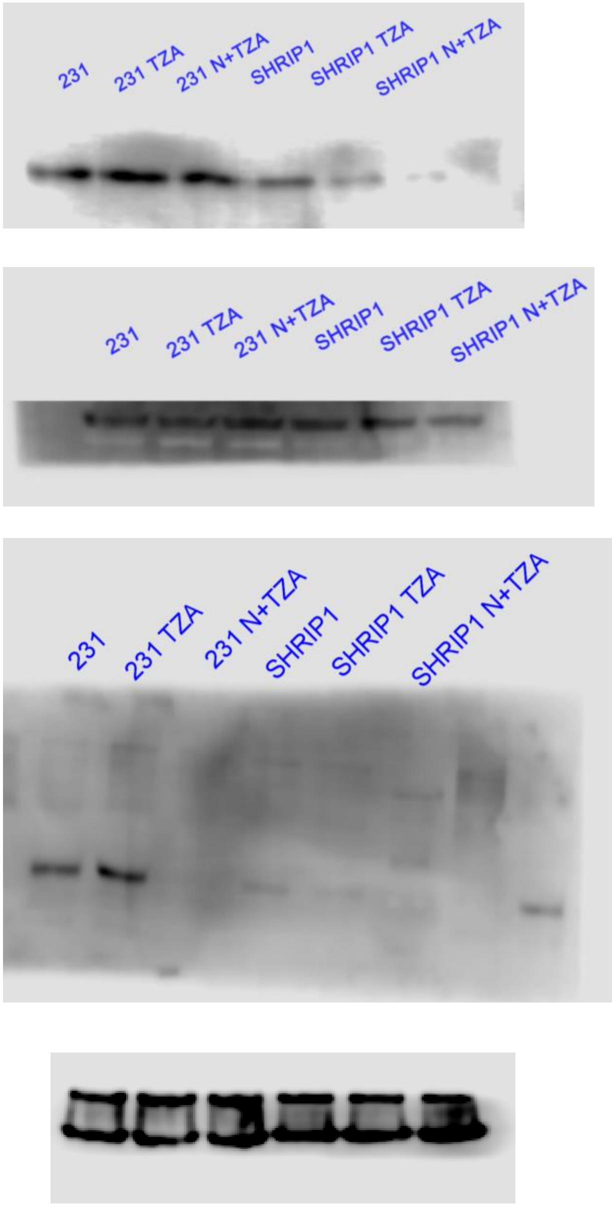

Figure 2C

eIF4E 28KD

AKT 55KD

p-AKT 60KD

GAPDH 37KD

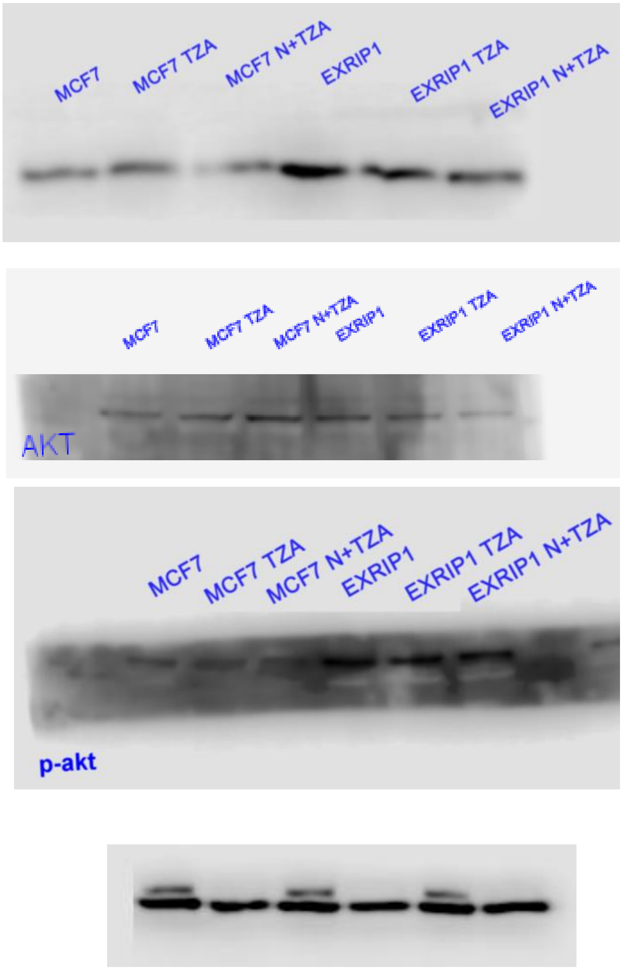

Figure 2E

eIF4E 28KD

AKT 55KD

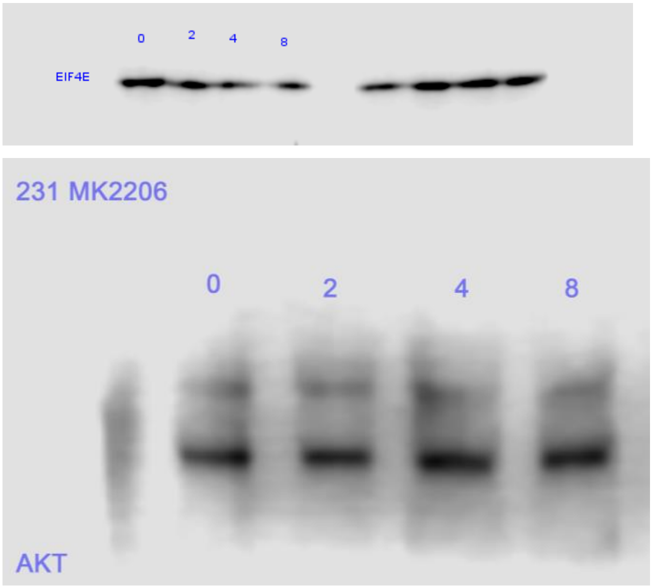

p-AKT 60KD

GAPDH 37KD

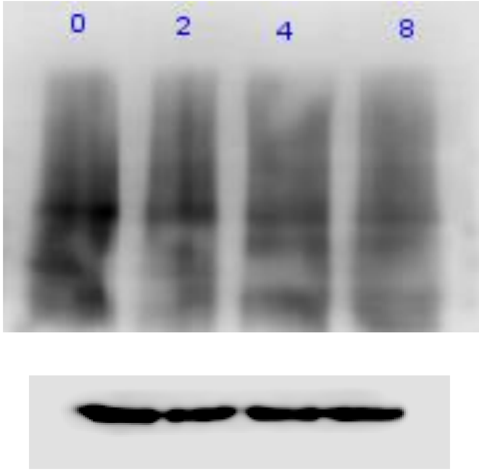

Figure 2F

eIF4E 28KD

AKT 55KD

p-AKT 60KD

GAPDH 37KD

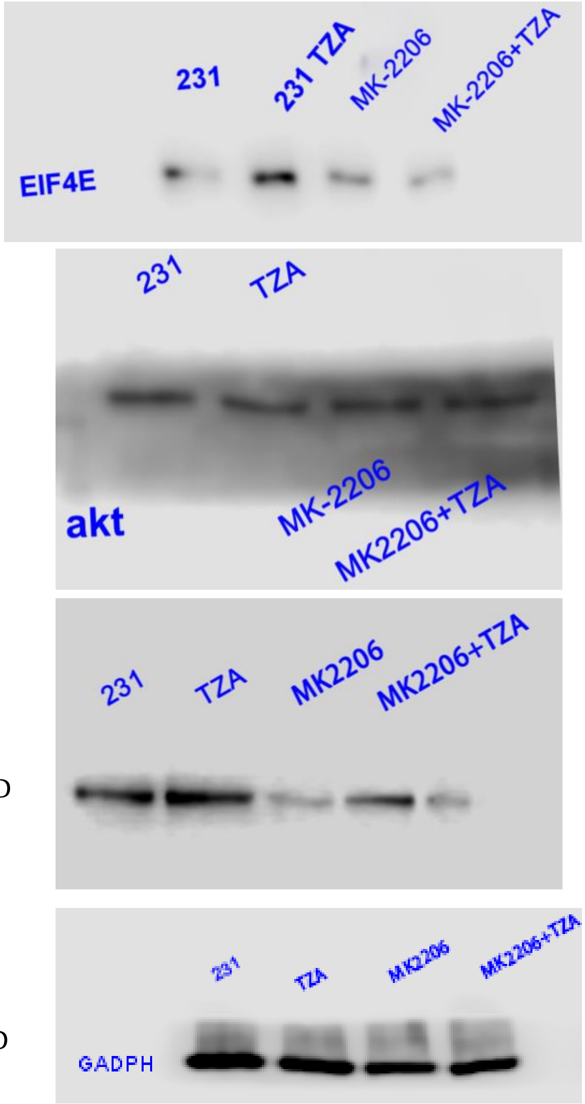

Figure 3I

E-cadherin 135KD

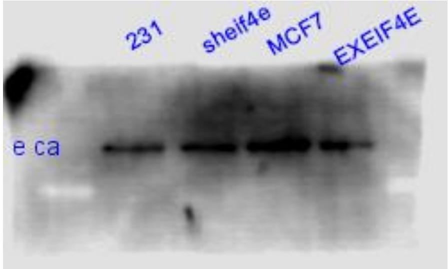

Vimentin 57KD

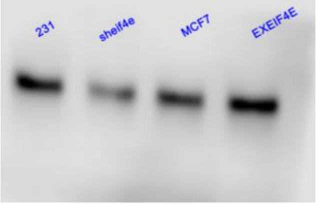

Snail 29KD

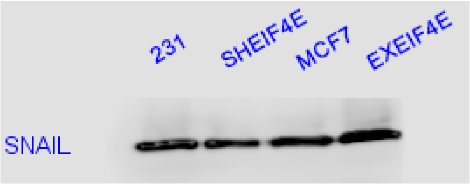

eIF4E 28KD

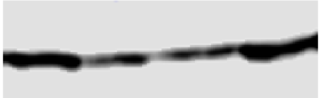

GAPDH 37KD

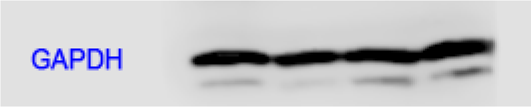

Figure 5A

MMP2 72KD

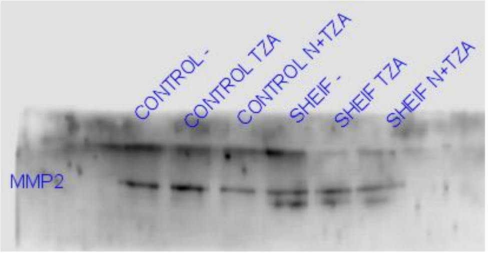

eIF4E 28KD

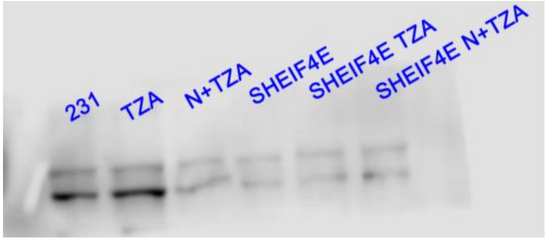

GAPDH 37KD

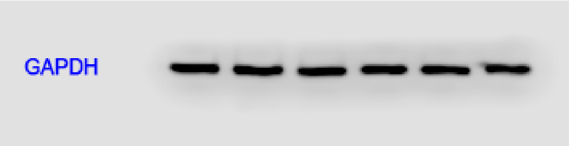

Figure 5C

eIF4E 28KD →

MMP2 72KD

GAPDH 37KD

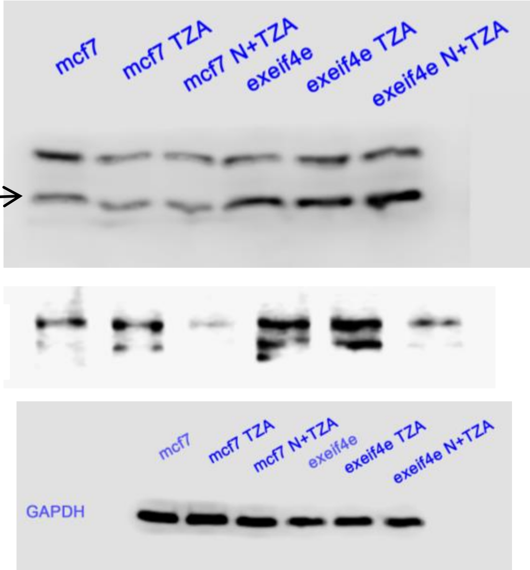

Figure S1A

RIPK1 76KD

GAPDH 37KD

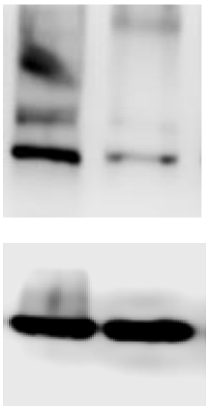

Figure S1C

RIPK1 76KD

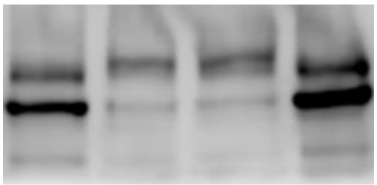

GAPDH 37KD →

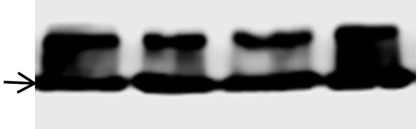

Figur S3A

MDA-MB-231 - T TZ ZA TZA

RIPK1 76KD

VE-cadherin 115KD

p-AKT 60KD

eIF4E 28KD

GAPDH 37KD

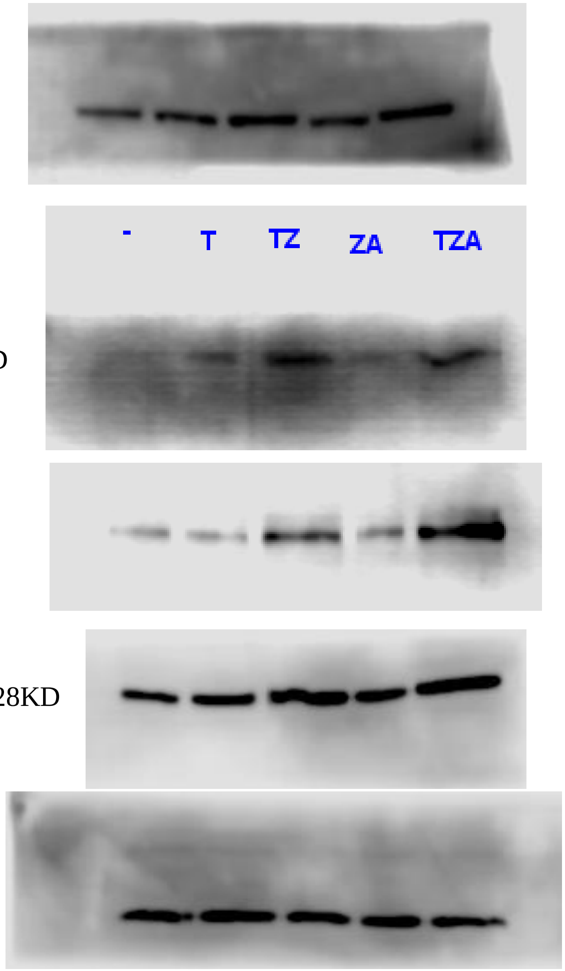

Figur S3B

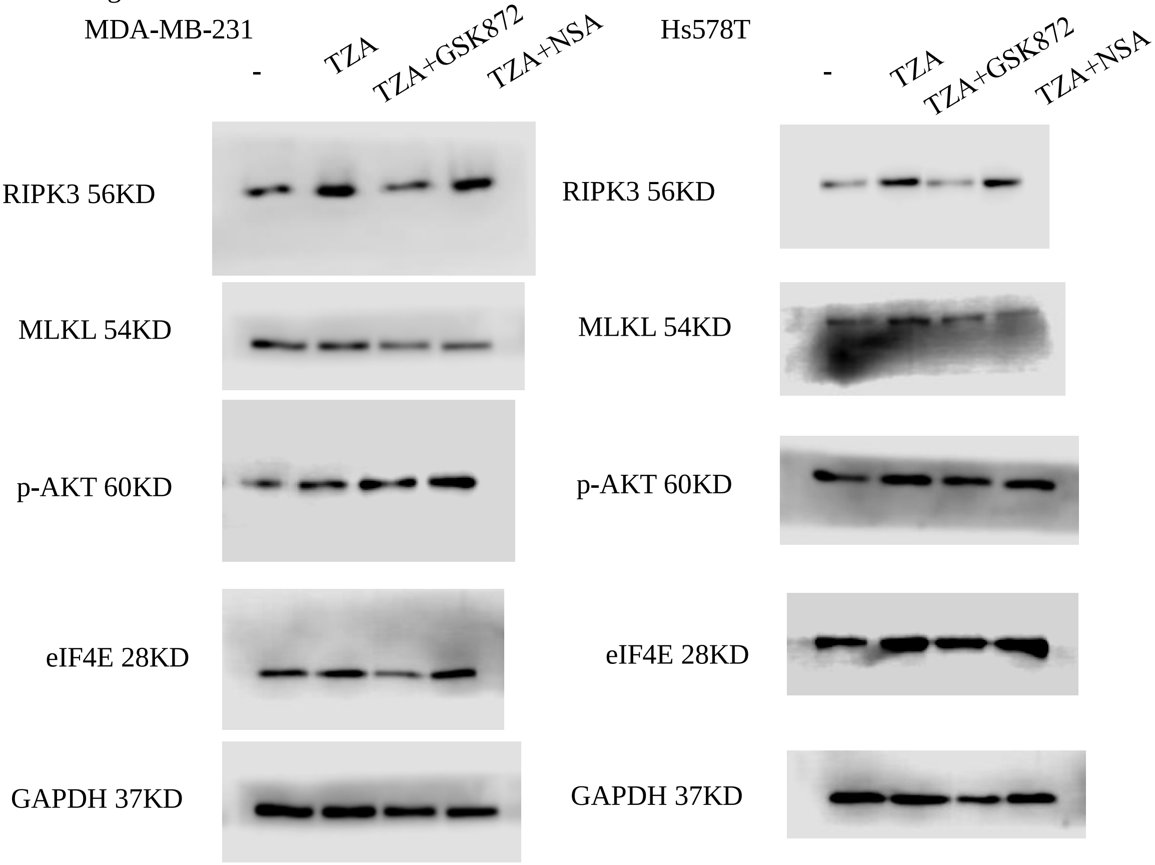

Figure S4A

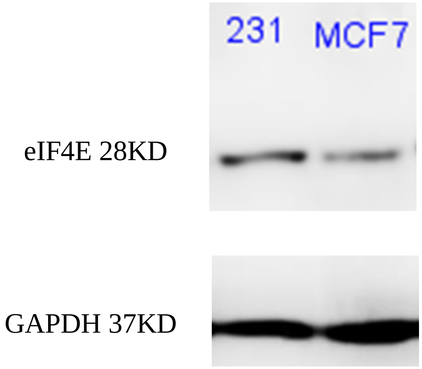

Figure S4C

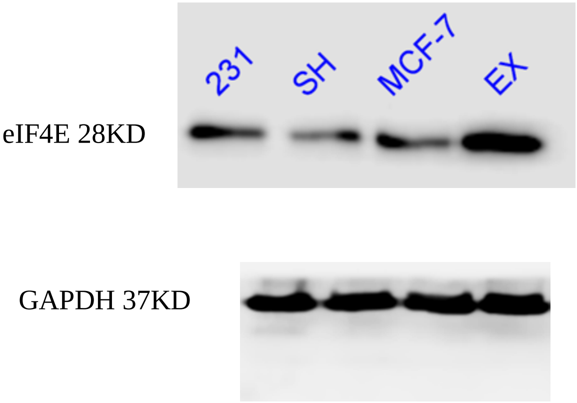

Supplement: Supplementary file 10 — Original Data File [file 41419_2023_5841_MOESM10_ESM.pdf]
